# Correlating Electronic Structure and Device Physics with Mixing Region Morphology in High‐Efficiency Organic Solar Cells

**DOI:** 10.1002/advs.202104613

**Published:** 2022-01-12

**Authors:** Shifeng Leng, Tianyu Hao, Guanqing Zhou, Lei Zhu, Wenkai Zhong, Yankang Yang, Ming Zhang, Jinqiu Xu, Junzhe Zhan, Zichun Zhou, Jiajun Chen, Shirong Lu, Zheng Tang, Zhiwen Shi, Haiming Zhu, Yongming Zhang, Feng Liu

**Affiliations:** ^1^ School of Chemistry and Chemical Engineering Frontiers Science Center for Transformative Molecules Shanghai Jiao Tong University Shanghai 200240 P. R. China; ^2^ School of Physics and Astronomy Shanghai Jiao Tong University Shanghai 200240 P. R. China; ^3^ Chongqing Institute of Green and Intelligent Technology Chongqing School University of Chinese Academy of Sciences (UCAS Chongqing) Chinese Academy of Sciences Chongqing 400714 P. R. China; ^4^ Center for Advanced Low‐dimension Materials State Key Laboratory for Modification of Chemical Fibers and Polymer Materials College of Materials Science and Engineering Donghua University Shanghai 201620 P. R. China; ^5^ Department of Chemistry Zhejiang University Hangzhou 310027 P. R. China

**Keywords:** electronic structure, energy loss, morphology, organic solar cells, photophysical process

## Abstract

The donor/acceptor interaction in non‐fullerene organic photovoltaics leads to the mixing domain that dictates the morphology and electronic structure of the blended thin film. Initiative effort is paid to understand how these domain properties affect the device performances on high‐efficiency PM6:Y6 blends. Different fullerenes acceptors are used to manipulate the feature of mixing domain. It is seen that a tight packing in the mixing region is critical, which could effectively enhance the hole transfer and lead to the enlarged and narrow electron density of state (DOS). As a result, short‐circuit current (*J*_SC_) and fill factor (FF) are improved. The distribution of DOS and energy levels strongly influences open‐circuit voltage (*V*_OC_). The raised filling state of electron Fermi level is seen to be key in determining device *V*_OC_. Energy disorder is found to be a key factor to energy loss, which is highly correlated with the intermolecular distance in the mixing region. A 17.53% efficiency is obtained for optimized ternary devices, which is the highest value for similar systems. The current results indicate that a delicate optimization of the mixing domain property is an effective route to improve the *V*_OC_, *J*_SC_, and FF simultaneously, which provides new guidelines for morphology control toward high‐performance organic solar cells.

## Graphical abstract

A tight packing in the mixing region effectively enhances the hole transfer and leads to the enlarged and narrow electron density of state. The optimized electronic structure effectively increases carrier density and reduces the recombination losses due to the reduced energy disorder, improving the open‐circuit voltage, short‐circuit current and fill factor simultaneously.

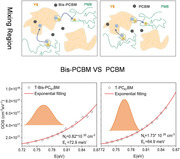

## 1 Introduction

The morphology of bulk heterojunction (BHJ) blends plays an important role in the performance of organic photovoltaic (OPV) devices.^[^ ^1^ ^]^ Attention has been paid to the phase separation and material crystallization in BHJ thin film,^[^ ^1^, ^2^ ^]^ which dictate the charge generation and carrier transport,^[^ ^1^, ^2^, ^3^ ^]^ leaving the mixing region property unexplored. In many cases, especially in high‐efficient non‐fullerene systems, the material interaction is similar, nevertheless, quite different device performances are recorded, indicating the mixing region can be of different features that are though amorphous.^[^ ^2^, ^3^, ^4^ ^]^ A full spectrum understanding of the thin film morphology requires a thorough investigation of the crystalline domain properties as well as the amorphous region characters, which in combination govern the carrier generation, transport, recombination, and energy loss. However, few relevant studies have comprehensively understood the relationship between the morphology of the mixing domain and the device performance. The introduction of a third component is an effective approach to optimize the morphology of the donor (D): acceptor (A) mixing region.^[^ ^5^ ^]^ For example, Hou et al. added [6,6]‐phenyl‐C71‐butyric acid methyl ester (PC_71_BM) in an all‐small‐molecule system that helped to form appropriate phase separation by adjusting the crystallization of non‐fullerene acceptors, achieving sufficient exciton dissociation.^[^ ^5d^ ^]^ Huang et al. found that the addition of TPE:4PDI (tetraphenylethylene core connect with four perylenediimide units) would induce more intermixed phases in a PTB7‐Th (poly[4,8‐bis(5‐(2‐ethylhexyl)‐ thiophene‐2‐yl)benzo[1,2‐b;4,5‐b′]dithiophene‐2,6‐diyl‐alt‐(4‐(2‐ethylhexyl)‐3‐fluorothieno[3,4‐b]thiophene)‐2‐carboxylate2‐6‐diyl]):ITIC‐Th (3,9‐bis(2‐methylene‐(3‐(1,1‐dicyanomethylene)‐indanone))‐5,5,11,11‐tetrakis(5‐hexylthienyl)‐dithieno[2,3‐d:2′,3′‐d′]‐s‐indaceno[1,2‐b:5,6‐b′]dithiophene) blended film, resulting in optimized phase separation which improved charge transfer.^[^ ^5b^ ^]^ Ye et al. added PC_71_BM to PM6 (poly[(2,6‐(4,8‐bis(5‐(2‐ethylhexyl‐3‐fluoro)thiophen‐2‐yl)‐benzo[1,2‐b:4,5‐b′]dithiophene))‐alt‐(5,5‐(1′,3′‐di‐2‐thienyl‐5′,7′‐bis(2‐ethylhexyl)benzo[1′,2′‐c:4′,5′‐c′]dithiophene‐4,8‐dione)]):N3 (2,2″‐((2Z,2″Z)‐((12,13‐bis(3‐ethylheptyl)‐3,9‐diundecyl‐12,13‐dihydro‐[1,2,5]thiadiazolo[3,4‐e]thieno[2″,3″:4″,5″]thieno[2″,3″:4,5]pyrrolo[3,2‐g]thieno[2″,3″:4,5]thieno[3,2‐b]indole‐2,10‐diyl)bis(methanylylidene))bis(5,6‐difluoro‐3‐oxo‐2,3‐dihydro‐1H‐indene‐2,1‐diylidene))dimalononitrile) system. With the reduction of non‐fullerene‐rich phase and PC_71_BM‐rich domain, a reasonable phase separation size was obtained at 20% PC_71_BM, which was conducive to exciton diffusion and device efficiency.^[^ ^5c^ ^]^ Although diverse in experiments, it is apparent that manipulating the mixing region morphology and electronic structure is critical in understanding the physical processes such as exciton dissociation and energy loss.

In this study, we study the mixing domain in the classic high‐performance PM6:Y6 (2,2′‐((2Z,2′Z)‐((12,13‐bis(2‐ethylhexyl)‐3,9‐diundecyl‐12,13‐dihydro‐[1,2,5]thiadiazolo[3,4‐e]thieno[2″,3″:4′,5′]thieno[2′,3′:4,5]pyrrolo[3,2‐g]thieno[2′,3′:4,5]thieno[3,2‐b]indole‐2,10‐diyl)bis(methanylylidene))bis(5,6‐difluoro‐3‐oxo‐2,3‐dihydro‐1H‐indene‐2,1‐diylidene))dimalononitrile) blends. Modified fullerene acceptors (FAs) have been used to manipulate the properties of the mixing domain. The high electron affinity, good electron transport capability, and low crystallinity make FAs ideal in manipulating the electron‐accepting properties of the mixing domain. Mono‐substituted FAs (Mono‐FAs) including [6,6]‐C61‐dimethyl 5‐methylisophthalate (C_61_DMI) and [6,6]‐phenyl‐C61‐butyric acid methyl ester (PC_61_BM)) and bis‐substituted FAs (Bis‐FAs) including [60] PCBM bis‐adduct (Bis‐PC_61_BM) and indene C60 bis‐adduct (IC_61_BA) are used. It is seen that different FAs could change the mixing domain properties significantly in packing density, charge transfer efficiency, and carrier diffusion. In detail, with the addition of Monos‐FAs, tighter packing in the mixing region is obtained. The hole/electron transfer among the amorphous components is improved. And the density of state (DOS) became higher with narrower distributions. Thus the optimized electronic structure leads to increased carrier density and reduced recombination losses. A 17.53% efficiency was obtained, which is a significant jump compared with those of binary blends (16.44%). These results spotlight the physical nature of the mixing region, which bears unique importance in dictating the electronic structure of the BHJ thin film, the optimization of which offers an important approach in obtaining higher performance OPV devices.

## 2 Results and Discussion

**Figure 1:**
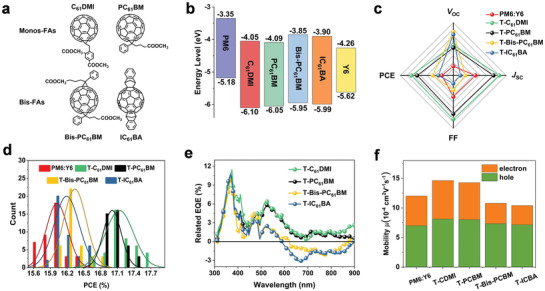
a) The chemical structures of C_61_DMI, PC_61_BM, Bis‐PC_61_BM, IC_61_BA. b) The energy levels of PM6, Y6, and four FAs. c) Radar map of photovoltaic parameters d) histogram of PCE measurement for 40 devices for PM6:Y6 based binary and ternary devices. e) Relative EQE curves of PM6:Y6 ternary devices compared to binary devices. f) Hole mobility and electron mobility of binary and ternary films.

**Figure** **1**a,b shows the chemical structures and the energy levels of materials used in this study. The highest occupied molecular orbital (HOMO) levels are measured by the ultraviolet photoelectron spectroscopy (UPS),^[^ ^6^ ^]^ and the lowest unoccupied molecular orbital levels (LUMO) are obtained by subtracting the HOMO with the optical band gaps (*E*_g_, Figures S1 and S3, Supporting Information).^[^ ^2a^ ^]^ Figure S2 (Supporting Information) shows the UV–vis absorption spectra of the binary and ternary BHJ films. The *E*_g_s were obtained by using the semiconductor *E*_g_ fitting method (Figure S4, Supporting Information).^[^ ^7^ ^]^ Binary and ternary OPV devices were fabricated with the device configuration of ITO/PEDOT:PSS/active layer/PFN‐Br/Al. The weight ratios of PM6:Y6 and PM6:Y6:FAs are 1:1.2 and 1:1.2:0.2, respectively. The photovoltaic parameters are compiled in **Table** **1** and Figure 1c. PM6:Y6 devices showed a maximum Power Conversion Efficiency (PCE) of 16.44%, with a *V*_OC_ of 0.827 V, a *J*_SC_ of 26.10 mA cm^−2^, and an FF of 76.1%. For PM6:Y6:Mono‐FAs ternary devices (T‐Mono‐FAs devices), the simultaneous enhancement of open‐circuit voltage (*V*_OC_), short‐circuit current (*J*_SC_), and fill factor (FF) is obtained. PM6:Y6:C_61_DMI ternary devices (T‐C_61_DMI devices) showed a maximum PCE of 17.53% with a *V*_OC_ of 0.848 V, a *J*_SC_ of 26.61 mA cm^−2^, and an FF of 77.70%. For T‐Bis‐FAs devices, the *V*_OC_ improved while *J*_SC_ and FF decreased. Figure S5 (Supporting Information) shows the external quantum efficiency (EQE) spectra of OPV devices, and the EQE variations of the ternary blends referencing PM6:Y6 binary blends are shown in Figure 1d. For PM6:Y6:Bis‐FAs ternary devices (T‐Bis‐FAs devices), the EQE in 300–400 and 500–600 nm regions gets improved slightly, which is attributed to the FAs absorption and better carrier extraction capability from the donor materials. The EQE from 600–900 nm accredited to Y6 absorption decreases significantly which causes a lower *J*_SC_. On the contrary, the EQE for T‐Mono‐FAs devices increases in the full spectrum region, suggesting a more efficient exciton splitting and carrier transport in ternary blends. Such effect is accredited to a better mixing morphology that both PM6 and Y6 excitons can be effectively extracted. It also indicates a better crystalline/mixing domain interface is formed to yield high carrier generation. The mobilities extracted by the space‐charge‐limited‐current (SCLC) method^[^ ^8^ ^]^ are shown in Figure 1f. For T‐Mono‐FAs films, balanced and high carrier transport is achieved (T‐C_61_DMI: *μ*_h_(hole mobility) / *µ*_e_(electron mobility) = 1.25; T‐PC_61_BM: *μ*_h_/*µ*_e_ = 1.28). On the contrary, T‐Bis‐FAs films show increased hole mobility and the decreased electron mobility (T‐Bis‐PC_61_BM: *μ*_h_/*µ*_e_ = 2.12; T‐IC_61_BA: *μ*_h_/*µ*_e_ = 2.20), causing reduced device FF.

**Table 1:** Optimal parameters of binary and ternary PSC devices

Blend	*V*_OC_ [V]	*J*_SC_ [mA cm^−^^2^]	FF [%]	PCE [%]^a)^
PM6:Y6	0.827 0.826 ± 0.002	26.10 25.80 ± 0.20	76.1 75.1 ± 0.7	16.44 16.00 ± 0.20
T‐C_61_DMI	0.848 0.846 ± 0.001	26.61 26.37 ± 0.23	77.7 76.8 ± 0.9	17.53 17.13 ± 0.25
T‐PC_61_BM	0.846 0.845 ± 0.002	26.51 26.26 ± 0.28	77.4 76.6 ± 0.7	17.36 17.00 ± 0.16
T‐Bis‐PC_61_BM	0.858 0.857 ± 0.002	25.87 25.64 ± 0.21	75.6 74.3 ± 0.8	16.78 16.33 ± 0.21
T‐IC_61_BA	0.856 0.854 ± 0.003	25.76 25.56 ± 0.16	75.1 74.1 ± 0.9	16.56 16.17 ± 0.22

^a)^ The average efficiency is obtained from 40 devices.

**Figure 2:**
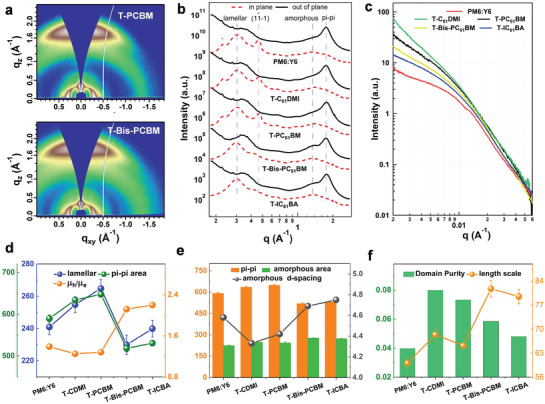
a) 2D GIWAXS patterns for T‐C_61_DMI and T‐IC_61_BA films. b) Scattering profiles for PM6:Y6 based binary and ternary films. c) RSoXS *I*‐*q* profiles d) peak area for pi–pi and lamellar diffraction peak and *μ*_h_/*µ*_e_ in binary and ternary blends. e) Fitting parameters of amorphous peaks. f) The intensity and length scale for fitting RSoXS profiles.

Grazing incidence wide‐angle X‐ray scattering (GIWAXS) was used to investigate the crystalline morphology of BHJ thin films.^[^ ^1^, ^2^ ^]^ **Figure** **2**a,b shows the 2D diffraction pattern and the 1D line‐cut profiles. The PM6:Y6 film shows similar crystalline properties to previous reports.^[^ ^2a,b^ ^]^ In brief, the (020) diffraction peak at ≈0.24 Å^−1^ and the (11−1) diffraction peak at ≈0.4 Å^−1^ in the in‐plane (IP) direction correspond to the Y6 long axis and body center cubic assembly induced by pi‐pi stacking. The lamellar stacking at ≈0.3 Å^−1^ and the pi‐pi stacking at ≈1.8 Å^−1^ come from both PM6 and Y6. An amorphous halo is seen in 1.3–1.5 Å^−1^ that summarizes the localized structure in the mixing region. The fitting results for these crystal planes are summarized in Table S1 (Supporting Information). In T‐Mono‐FAs blends, the Y6 (020) and (11−1) peaks are maintained with slightly decreased peak area and crystal coherence length (CCL). In T‐Bis‐FAs blends, the (020) peak disappears, the CCL and peak area of Y6 (11−1) peak also decrease significantly. Figure 2d shows the crystallinity changes of the pi‐pi stacking and the lamellar peaks for PM6 and Y6, which increase simultaneously in Mono‐FA blends but decrease in Bis‐FA blends. The enhanced crystallinity in T‐Mono‐FAs blends accounts for the carrier mobility improvement. The amorphous peak information is shown in Figure 2e (OOP direction fitting, compared with pi‐pi stacking). Combined with the results of (020) and (11−1) peaks, the increased amorphous peak area and reduced pi‐pi stacking in T‐Bis‐FAs films demonstrate the deterioration of Y6 crystals, which originates from the good mixing between Y6 and Bis‐FAs (Figures S10–S12, Supporting Information). The poor Y6 crystallization causes reduced electron mobility, leading to unbalanced carrier mobility. Unbalanced carrier mobility impedes efficient carrier transport, resulting in a decrease in FF. The d‐spacing of the amorphous peak (*d*_ap_) in five blends (PM6:Y6, PM6:Y6:CDMI (T‐CDMI), PM6:Y6:PCBM (T‐PCBM), PM6:Y6:Bis‐PCBM (T‐Bis‐PCBM), PM6:Y6:ICBA (T‐ICBA)) is 4.58, 4.33, 4.42, 4.69, and 4.75 Å, respectively. This result indicates an important feature in the mixing region, where Mono‐FAs could enhance the average molecular distance, and the Bis‐FAs works oppositely. This distance change, although small in value, is key in dictating the electronic structure of the mixing domain. A tighter intermolecular packing could facilitate exciton splitting and carrier diffusion outwards to a crystalline domain under the charge separation and hopping transport theory framework.

Resonant soft X‐ray scattering (RSoXS) was used to investigate the phase separation of BHJ thin films.^[^ ^9^ ^]^ In RSoXS at off‐edge energies, the scattering contrast comes from the electron density variation between crystalline and amorphous regions in PM6:Y6 blends. When FAs are added, the unique carbon K‐edge absorption from fullerene can be utilized.^[^ ^9^ ^]^ In ternary blends, the fullerenes are dispersed in the amorphous mixing region, which acts as a tracer in revealing the mixing domain features. The RSoXS I‐q profiles are shown in Figure 2c, and the analytic results are shown in Figure 2f. The correlation length model is used to extract the correlation length of domains. The phase purity is obtained by integrating Iq^[^ ^2^ ^]^ over the q range since the major concern for the current system is to look into the fullerene incorporated mixing domain and crystalline phase differences.^[^ ^2^, ^4^ ^]^ In comparison with PM6:Y6, T‐Mono‐FAs blends show slightly enhanced correlation lengths, while T‐Bis‐FA blends show largely increased correlation lengths. The phase purity decreases from T‐Mono‐FAs blends to T‐Bis‐FAs blends. These results reveal the different morphology in BHJ blends regarding the mixing domain. The addition of Mono‐FAs does not significantly enlarge the mixing domain size but induces intimate mixing with the other amorphous contents in blends. Bis‐FAs in blends dilate the mixing domain and enlarge the intermolecular distances. The higher phase purity in T‐Mono‐FAs blends further confirms high electron density in the mixing region, and thus the advantage of its electronic properties can be utilized. Such a feature plays an important role in device operation since the D/A mixture governs charge transfer kinetics and energy loss of the BHJ thin film.

**Figure 3:**
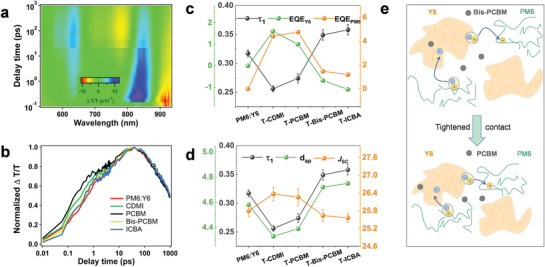
a) Color plot of fs transient absorption spectra of PM6:Y6:IC_61_DMI blend film and b) TA kinetics of PM6:Y6 based binary and ternary blend showing the hole transfer process. c) The relationship among *τ*_1_(hole transfer time), EQE_PM6_, and EQE_Y6_. d) The relationship among *τ*_1_, *J*_SC_ and dap (amorphous d‐spacing). e) Schematic diagram of hole/electron transfer for T‐Mono‐FAs and T‐Bis‐FAs blends.

We then investigate the charge‐transfer process in BHJ thin films by femto‐second transient absorption spectroscopy (TAS) and time‐resolved photoluminescence spectrum (TRPL) measurements.^[^ ^10^ ^]^ A 750 nm excitation was used to pump the Y6 acceptor only to study the Y6 to PM6 hole‐transfer. A representative 2D color plot of TAS (PM6:Y6:C_61_DMI) is shown in **Figure** **3**a and the representative TAS profiles at indicated delay times are shown in Figures S17–S19 (Supporting Information). It is seen that with the decay of the Y6 bleach peak in 650–900 nm, a bleach peak at 540–650 nm rises that corresponds well with the PM6 absorption. The Y6 bleach decay agrees well with the PM6 bleach rising process, confirming the hole transfer from Y6 to PM6 (Figure S20, Supporting Information). Figure 3b shows the hole‐transfer dynamics by the biexponential fit of the ≈590 nm bleach signal. The fast components (*τ*_1_) represent the ultrafast exciton dissociation in the mixing region,^[^ ^8^, ^11^ ^]^ which are ≈0.32, ≈0.25, ≈0.27, ≈0.35, and ≈0.36 ps, for PM6:Y6, T‐CDMI, T‐PCBM, T‐Bis‐PCBM, T‐ICBA. This result corresponds well with the intermolecular distances in the mixing region. TRPL is used to investigate the electron transfer from PM6 to Y6 using a 480 nm laser excitation and 500–650 nm probing region.^[^ ^2a^ ^]^ As shown in Figure S21 (Supporting Information), ternary blends show faster electron transfer than that of binary blends, and Mono‐FAs are more efficient in driving this action (PM6:Y6: 89.42 ps, T‐C_61_DMI: 80.41 ps, T‐PC_61_BM: 83.45 ps, T‐Bis‐PC_61_BM:85.67 ps, T‐IC_61_BA:87.89 ps). The faster electron transfer could be related to the better electron–extraction capacity of fullerenes. The multi‐variable correlations regarding charge transfer, segmental EQE responses, *J*_SC_, and intermolecular distances are summarized in Figure 3c,d. The hole–transfer kinetics shows a high correlation with the intermolecular distances in the mixing region. It should be noted that fullerene is a good electron‐accepting and hole blocking material, but its addition in the mixing region does not disturb the hole transfer from Y6 to PM6. Thus not only the intermolecular distance but also closer Y6 to PM6 contact existed in T‐Mono‐FAs blends. The integrated EQE responses for Y6 and PM6 in their responsive regions are calculated and shown in Figure 3c. For T‐Monos‐FAs blends, both Y6 and PM6 show EQE improvements. For T‐Bis‐FAs blends, EQE_PM6_(EQE contributed by PM6) shows a slight increase and EQE_Y6_(EQE contributed by Y6) shows a negative response, indicating the hole‐extraction in the mixing domain is affected. The *J*_SC_ correlates well with the hole‐transfer kinetics and EQE_Y6_, indicating the Y6 carrier generation contributes significantly to J_SC_. Schemes regarding the mixing region in ternary blends are shown in Figure 3e,f, where we show that the close packing in T‐Mono‐FAs blends is the fundamental reason that helps to improve the charge transfer and transport, which is cross‐validated by the negative effect in T‐Bis‐FAs blends. Exciton dissociation efficiencies are evaluated by *J*_ph_(photocurrent)–*V*_ef_(effective voltage) analysis^[^ ^3c^ ^]^ (Figure S22, Supporting Information), yielding values of 95.7%, 98.1%, 97.5%, 94.5%, 94.2% for PM6:Y6, T‐CDMI, T‐PCBM, T‐Bis‐PCBM, and T‐ICBA blends, which further support the observations of the mixing region characteristics.

**Figure 4:**
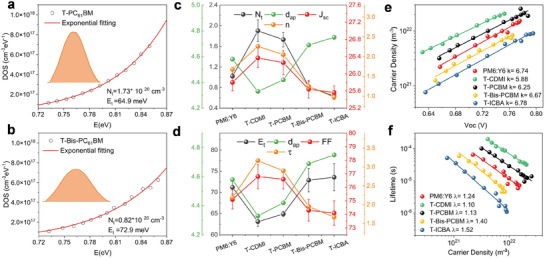
The electron DOS curves for a) T‐PCBM devices and b) T‐Bis‐PCBM devices. c) The relation of *N*_t_ (DOS density), *d*_ap_ (amorphous d‐spacing), *n* (carrier density), and *J*_SC_. d) The relation of *d*_ap_, *E*_t_ (the energetic energy for describing energy disorder), *τ* (carrier lifetime) and FF. e) Density charge‐carriers under different *V*_OC_ conditions. f) Charge lifetime in the devices as a function of carrier density.

The amorphous mixing region plays an important role in charge transfer, carrier diffusion, and recombination. We then look into the electronic structure density of state (DOS) of the BHJ thin films that best summarize the key electronic properties. The distribution of electron DOS is described by an exponential distribution in organic semiconductors:^[^ ^12^ ^]^

(1) $$g_{n}\left(E\right)\left[{\mathrm{eV}}^{-1}\cdot {\mathrm{cm}}^{-3}\right]=\frac{N_{t}\left[{\mathrm{cm}}^{-3}\right]}{E_{t}\left[\mathrm{eV}\right]}\;\;\exp\left[-\frac{E-E_{\mathrm{LUMO}}\left[\mathrm{eV}\right]}{E_{t}\;\left[\mathrm{eV}\right]}\right]$$

where *N*_t_ is the total density per unit volume, *E*_LUMO_ is the LUMO energy level, and *E*_t_ is the energetic energy for exponential tail distribution that describes energy disorder. Impedance Spectroscopy (IS) is employed to access the DOS information. The chemical capacitors ($C_{\mu }^{n}$) determined by IS reflect the capability of the photovoltaic device to accept or release additional charge carriers as the result of the shifting in the quasi‐Fermi level *E*_Fn_.^[^ ^13^ ^]^ As the quasi‐Fermi level (*E*_Fn_) reflects the electron filling level. Using the zero‐temperature approximation of Fermi function at occupancy >1%, DOS *g*_n_(*E*_Fn_) is obtained by extracting the $C_{\mu }^{n}$ following the relationship:^[^ ^13^, ^14^ ^]^

(2) $$C_{\mu }^{n}\left[F\cdot cm^{-3}\right]=Lq^{2}g_{n}\left(E_{\mathrm{Fn}}\right)\left[cm^{-3}\cdot eV^{-1}\right]$$

where *L* is the thickness of the active layer, *q* is the elemental charge. By applying different illumination and corresponding the bias voltage, the quasi‐Fermi level moves across the electronic states, and the scatter plot of the filling state as a function of voltage can be obtained. The exponential fitting results for binary and ternary devices are shown in **Figure** **4**a,b, and Figure S23 (Supporting Information), and the fitting parameters are summarized in Table S3 (Supporting Information). Larger *N*_t_ (1.73, 1.90 *10^16^ cm^−3^ eV^−1^) and smaller *E*_t_ (64.9, 63.1 meV) are obtained for T‐PCBM and T‐CDMI devices compared to that in PM6:Y6 devices (*N*_t_ = 1.02*10^16^ cm^−3^ eV^−1^; *E*_t_ = 71.2 meV), suggesting a higher and narrower DOS are formed with the addition of Mono‐FAs. The relationship among DOS, morphology, and device performance is summarized in Figure 4c,d. It is seen that *N*_t_ and *J*_SC_ are well correlated, which are inverse to amorphous inter‐molecular distance (*d*_ap_), thus a tightly packed mixing domain contributes to a greater total electron density and *J*_SC_. This effect is similar to n‐type doping in blends by introducing Mono‐FAs. The energy disorder (*E*_t_) is highly correlated with the amorphous inter‐molecular distance. Larger energy disorder, widely distributed, and lower DOS are seen in T‐Bis‐FAs devices, which are caused by the loose packing in the mixing region since the energy disorder is determined by the structural order.^[^ ^15^ ^]^

The transient photovoltage (TPV) and transient photocurrent (TPC) measurements were used to investigate the carrier kinetic in OPV devices.^[^ ^16^ ^]^ As shown in Figure 4e and Figure S25 (Supporting Information), the carrier lifetimes (*τ*) and carrier density (*n*) under different *V*_OC_ conditions could be obtained using exponential function fitting. The T‐Mono‐FAs devices showed higher *τ* and *n* while the T‐Bis‐FAs devices exhibited lower *τ* and n compared to that of PM6:Y6 devices (Table S4, Supporting Information). The differential capacitance method is used to calculate the relation between *τ* and n (shown in Figure 4f). It is seen that the carrier lifetime (*τ*) as a function of change density (*n*) follows an approximated exponential law: $\tau \;=\tau_{0}\;{\left(\frac{n}{n_{0}}\right)}^{\lambda }$, suggesting that the non‐geminate recombination was the dominant loss mechanism.^[^ ^17^ ^]^ The recombination order *R* (*R* = *λ* + 1) was calculated by the exponential factor *λ* (*λ* equal to the slope of lg *τ* vs lg *n)*, *R* is expected to be 2 in strictly bimolecular recombination conditions^[^ ^17^, ^18^ ^]^, and a higher value of *R* (*R* > 2) measures the defects and inherent energy disorder that cause carrier recombination.^[^ ^17b^ ^]^ In T‐Mono‐FAs, *R* is close to 2 (T‐C_61_DMI: 2.13; T‐PC_61_BM: 2.10), and *R* is much larger in T‐Bis‐FAs devices (T‐IC_61_BA: 2.52; T‐Bis‐PC_61_BM: 2.40). Thus the Mono‐FAs blends are bimolecular recombination dominated systems, and the Bis‐FAs blends suffer from serious shallow trap induced recombination. Figure S26 (Supporting Information) shows the relationship between the non‐geminate recombination rate coefficient *k* (*n*) and charge density, where *k* (*n*) can be calculated by the equation: $k\left(n\right)\;=\frac{1}{\tau (n)\;\times n}\;$.^[^ ^19^ ^]^ The smallest recombination rate coefficient is seen in T‐Mono‐FAs, further confirming the recombination analysis.

The combination of DOS and carrier kinetics enables the analysis of the thin film electronic structure characteristics. As seen in Figure 4c, the variation of carrier density (*n*) agrees with the trends of *N*_t_, which cross‐validate the DOS and TPC/TPV measurement since $n_{n}=N_{t}\;\text{exp}\left(-\frac{E_{c}-E_{F}}{k_{0}T}\right)$, where *n*_n_, *E*_c_, and *E*_F_ are the electron density, the conduction band energy, and the Fermi level. It should be noted that the electron/hole transfer also affects the number of free charge carriers, whose kinetics is inversely related to carrier density (Figure 3d). The carrier density shows a similar trend with *J*_SC_ (Figure 4c), suggesting an important mechanism in dictating the device current. Another important factor that affects *J*_SC_ is the trap‐assisted recombination, which is strongly correlated with the energy disorder (*E*_t_, Figure 4d). It is also noticed that the carrier lifetime is inversely correlated with the energy disorder parameter (Figure 4d), which is a common practice since the localized carrier is easier to migrate toward or tunnel to the lower‐energy trap sites in broadened‐DOS in devices to induce recombination.^[^ ^15b^ ^]^ However, new insights are obtained in the variation of FF, which is in good correlation with carrier density and carrier lifetime, and thus opposed to the energy disorder variations and the packing distances. These results highlight the importance of the mixing region, where a close packing and a higher DOS can be of critical importance, which not only affects the *J*_SC_ but also FF, the device parameters that are believed to be strongly correlated with transport properties.

**Figure 5:**
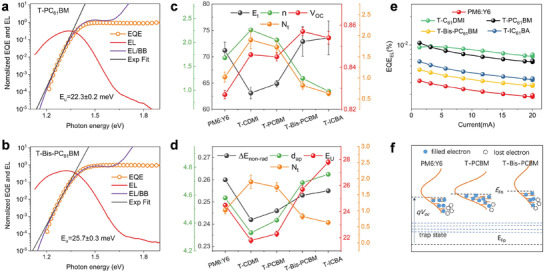
Highly sensitive EQE and EL for a) T‐PCBM, b) T‐Bis‐PCBM blended devices. c) The relationships among *E*_t_ (the energetic energy for describing energy disorder), *n* (carrier density), *V*_OC_ *, N*_t_ (DOS density). d) The relationships among *E*_U_ (Urbach energy), *∆E*_non‐rad_ (non‐radiative recombination loss), *d*_ap_ *, N*_t_ (DOS density). e) EQE_EL_, f) *V*_OC_ diagram for filling states and non‐radiative recombination loss.

The device *V*_OC_ is related to the electronic structure of the blended thin films, which is subject to energy loss (*E*_loss_). It is established that the charge transfer (CT) state is an important factor that dictates *E*_loss_. However, it is also reported that the CT state is quite weak in PM6:Y6 blends^[^ ^20^ ^]^ (no obvious charge‐transfer peaks were observed in **Figure** **5**a,b (highly sensitive external quantum efficiency (s‐EQE)), which persuades us to investigate the *V*_OC_ variation upon electronic structure change. The relationship among electronic structure, morphology, energy loss, and *V*_OC_ is summarized in Figure 5c,d. The DOS shape and height and the number of electrons would affect the state of electronic filling, the highest filling state quasi‐Fermi level (*E*_Fn_) determines *V*_OC_, as *V*_OC_ is determined by *E*_Fn_ – *E*_Fp_ (Taken *E*_Fp_ as a fixed value).^[^ ^14^, ^21^ ^]^ In the impedance measurement, the blended thin film is considered as a collection of unit capacitors, the charging and discharging processes reveal the fundamental electronic properties of the thin film. The FAs, either Mono‐substituted or Bis‐substituted ones have a slightly higher LUMO compared to Y6, whose addition in PM6:Y6 blends would tailor the band tail and changes the *E*_Fn_ to a slightly higher value, and thus giving rise to higher *V*_OC_s. Such effect is easy to understand in T‐Monos‐FAs blends since both DOS and carrier density are increased and slightly lower LUMO for PCBM. In T‐Bis‐FAs blends, less carrier density is recorded, which is accompanied by the simultaneously reduced DOS but with improved *V*_OC_s. This is due to the larger energy level splitting between Bis‐FAs and Y6, that the hybridization of Bis‐FAs and Y6 tail states host much fewer carriers and can reach a higher filling energy level (higher *E*_Fn_), thus higher *V*_OC_ is obtained. The energy disorder related to tail states can cause energy loss, which is analyzed in DOS and s‐EQE spectra (Figure 5a,b). Urbach energy (*E*_U_) was obtained by fitting the FTPS‐EQE,^[^ ^22^ ^]^ which characterizes the distribution of electrons in tail states correlating with energy disorder, a smaller *E*_U_ represents a smaller energy disorder. *E*_U_ for PM6:Y6, T‐CDMI, T‐PCBM, T‐Bis‐PCBM, T‐ICBA devices is 24.5, 21.8, 22.3, 25.7, and 27.8 meV, which show good agreement with *E*_t_ (Figure 4d). The non‐radiative recombination loss (Δ*E*_non‐rad_) is the commonly believed major loss channel in OPV devices, which is probed by the electroluminescence (EL) of solar cells. Figure 5e shows the EL quantum efficiency (EQE_EL_) profiles. The Δ*E*_non_ *_‐_*_rad_ can be obtained by Δ *E*_non − rad_ =   − kT*In(EQE_EL_).^[^ ^2a^ ^]^ *∆E*_non‐rad_ for PM6:Y6, T‐CDMI, T‐PCBM, T‐Bis‐PCBM, T‐ICBA devices are 0.260, 0.242, 0.246, 0.253, and 0.255 eV, respectively, whose trend is in good correlation with *E*_U_ and mixing region intermolecular distances (Figure 5d), suggesting the role of energy disorder in non‐radiative recombination energy loss. In a mixture with large energy disorder, shallow traps can cause increased non‐radiative recombination loss. However, we see *E*_U_ in T‐Bis‐FAs devices is slightly larger compared to PM6:Y6 blends, but the Δ*E*_non‐rad_ is smaller compared to binary devices. Such phenomenon is accredited to the lower intensity DOS tail for T‐Bis‐FAs blends that the tail state of the same energy is reduced (reduced *N*_t_, Figure 5d) compared to PM6:Y6 blends, and non‐radiative recombination probability is thus reduced. A schematic illustration regarding the *V*_OC_ and energy loss is shown in Figure 5f. The increased carrier density associated with the DOS shape change dictates the *E*_Fn_ and Δ*E*_non‐rad_, and in T‐Monos‐FAs blends, key parameters get improved, affording a higher *V*_OC_. These energy landscapes also affect the carrier kinetics, as visualized in TAS and TPC/TPV measurements. Thus optimizing the mixing state morphology and electronic structure can be a critical step to achieve high‐performance OPVs. In OPV blends, the crystalline morphology is well studied, and the current work tries to raise up the importance of the mixing domain properties, which could provide new insight in helping to fabricate high‐efficiency devices for new material systems.

## 3 Conclusion

To conclude, the electronic structure and morphology of the amorphous region in highly efficient OPV blends are investigated. We see that the proper choice of the doping agents plays an important role. With the addition of Mono‐FAs, tightly condensed amorphous structures are formed, which effectively promotes the carrier transfer and increases the total electron DOS, obtaining high carrier density. The tightly condensed mixing domain largely reduces the energy disorder, which effectively increases the carrier lifetime and reduces recombination loss since fewer carriers can be trapped to induce unfavorable pathways. These features help to improve *J*_SC_ and FF in OPV devices. The increased carrier density with reduced energy disorder favor electrons to fill to higher quasi‐Fermi levels, accounting for a higher *V*_OC_. While in the previous research large efforts have been devoted to understanding the crystalline morphology in BHJ blends, this work argues for the importance of the mixing domain, which provides a different avenue in manipulating the device characters to achieve higher efficiency.

## 4 Experimental Section

See experimental details in the Supporting Information.

## Conflict of Interest

The authors declare no conflict of interest.

## Supporting information

Supporting Information

## Data Availability

The data that support the findings of this study are available from the corresponding author upon reasonable request.
