# Supplementary material for: Correlating Electronic Structure and Device Physics with Mixing Region Morphology in High‐Efficiency Organic Solar Cells
Source: Adv Sci (Weinh). 2022 Jan 12;9(6):2104613. doi: 10.1002/advs.202104613 (PMC8867200; doi:10.1002/advs.202104613)
Supplement: Supplementary file 1 — Supporting Information [file ADVS-9-2104613-s001.pdf]

ADVANCED SCIENCE
Open Access

Supporting Information

for *Adv. Sci.*, DOI: 10.1002/advs.202104613

Correlating electronic structure and device physics with mixing region morphology in high-efficiency organic solar cells

*Shifeng Leng, Tianyu Hao, Guanqing Zhou, Lei Zhu, Wenkai Zhong, Yankang Yang, Ming Zhang, Jinqiu Xu, Junzhe Zhan, Zichun Zhou, Jiajun Chen, Shirong Lu, Zheng Tang, Zhiwen Shi, Haiming Zhu, Yongming Zhang, Feng Liu**

Supporting Information

Correlating electronic structure and device physics with mixing region morphology in high-efficiency organic solar cells

*Shifeng Leng, Tianyu Hao, Guanqing Zhou, Lei Zhu, Wenkai Zhong, Yankang Yang, Ming Zhang, Jinqiu Xu, Junzhe Zhan, Zichun Zhou, Jiajun Chen, Shirong Lu, Zheng Tang, Zhiwen Shi, Haiming Zhu, Yongming Zhang, Feng Liu**

S. Leng, T. Hao, G. Zhou, Dr. L. Zhu, Dr. W. Zhong, Dr. Y. Yang, M. Zhang, J. Xu, Z. Zhou, Prof. Y. Zhang, Prof. F. Liu
School of Chemistry and Chemical Engineering, Frontiers Science Center for Transformative Molecules, Shanghai Jiao Tong University, Shanghai 200240, P. R. China.
E-mail: fengliu82@sjtu.edu.cn

J. Zhan, J. Chen, Prof. Z. Shi
School of Physics and Astronomy, Shanghai Jiao Tong University, Shanghai 200240, P. R. China

Prof. S. Lu
Chongqing Institute of Green and Intelligent Technology, Chongqing School, University of Chinese Academy of Sciences (UCAS Chongqing), Chinese Academy of Sciences, Chongqing 400714, P. R. China

Prof. H. Zhu
Department of Chemistry, Zhejiang University, Hangzhou 310027,P. R. China

Prof. Z. Tang
Center for Advanced Low-dimension Materials, State Key Laboratory for

Modification of Chemical Fibers and Polymer Materials, College of Materials Science and Engineering, Donghua University, Shanghai 201620, P. R. China

Experimental Section and device characterization

General Methods The morphologies of the nanostructures were characterized by transmission electron microscopy (TEM, JEM-1400, JEOL, Japan). The GIWAXS characterization of the thin films was performed at the Advanced Light Source (Lawrence Berkeley National Laboratory) on beamline 7.3.3. The incidence angle was 0.16°, and the beam energy was 10 keV. Samples were prepared under device conditions on the Si substrates. R-SoXS was performed at beamline 11.0.1.2 (ALS, LBNL) with a bean energy of 285.0 eV. Samples were prepared under device conditions on the Si/PEDOT:PSS substrates, then placed in water and transferred to a silicon nitride window.

Chemicals and Reagents All reagents and chemicals were purchased from commercial sources (Aldrich or Acros) and used without further purification. PM6 and Y6 were purchased from Solarmer company. C_{61}DMI was syntehsized by Prof. Lu Shirong. $PC_{61}BM$, Bis-$PC_{61}BM$, $IC_{61}BA$ were purchased from American Dye Source, Inc.

Device Fabrication Organic solar cell devices with ITO/PEDOT:PSS/active layer/PFN-Br/Ag structures were fabricated. Patterned ITO glass substrates were sequential cleaned by ultrasonicating in acetone, detergent, deionized water and isopropyl alcohol for 15 min each and then dried under dry oven. The precleaned substrates were treated in an ultraviolet-ozone chamber for 15 min, then a ~40 nm thick PEDOT:PSS (Clevious PVP AI 4083 H. C. Stark, Germany) thin film was deposited onto the ITO surface by spin-coating and baked at 150 °C for 15 min. The active layer solution of PM6:Y6 (1:1.2, wt%, 6.5 mg mL^{-1} for PM6) in CF (with 0.5% CN solvent additive) and PM6:Y6:FAs (1:1.2:0.2, wt%, 6.5 mg mL^{-1} for PM6) in CF (with 0.5% CN solvent additive) were stirred at 25 °C for 2h, and then spin-coated on top of the PEDOT:PSS layer (2300 rpm,40s). The optimum thickness of the active layer is 140 nm. The prepared films were treated with thermal annealing at different temperature for 5 min. After cooling to room temperature, a ~5 nm thick of PFN-Br was spin-coated on the top of active layer. Then, those samples were brought into to an evaporate chamber and a 150 nm thick silver layer was thermally evaporated on the PFN-Br layer at a base pressure of 1×10^{-6} mbar. The evaporation thickness was controlled by SQC-310C deposition controller (INFICON, Germany). Twelve devices were fabricated on one substrate and the active area of each device was 0.05 cm^2

defined by a shadow mask.

Device Characterization. Device performance was measured by using a 510 Air Mass 1.5 Global (AM 1.5 G) solar simulator (SS-F5-3A, Enlitech) with an irradiation intensity of 100 mW cm^{-2}, which was demarcated by a calibrated silicon solar cell (SRC2020, Enlitech). The *J-V* characteristics were measured along the forward scan direction from -0.5 to 1 V, with a scan step of 50 mV and a dwell time is 10 ms using a Keithley 2400 Source Measure Unit. EQE spectra were measured by using a solar-cell spectral-response measurement system (QE-R3011, Enlitech).

Ultraviolet photoelectron spectroscopy measurement. Ultraviolet Photoelectron Spectroscopy analysis was conducted using an AXIS Ultra DLD spectrometer (Kratos Analytical Inc., Mancheser, UK) with a He discharge UV lamp with He I radiation (incident photo energy, 21.22 eV), a hemispherical analyzer operating in the fixed analyzer transmission mode and the standard aperture (analysis area: 0.3 mm * 0.7 mm).

SCLC Mobility Measurements. The electron-only devices were fabricated with ITO/ZnO/PM6:Y6/ZnO/Ag structures and hole-only devices were fabricated with ITO/PEDOT:PSS/PM6:Y6/MoOx/Al structures. The thickness of the active layer is 140 nm. The space charge limited current (SCLC) mobility was calculated according to the Mott-Gurney square law $J = 9\varepsilon_r\varepsilon_0\mu V^2/8L^3$, where J is the current density, ε_r is the relative dielectric constant of the transport medium component, ε0 is the vacuum permittivity, μ is the electron or hole mobility, V is the effective voltage, and L is the thickness of active layer.

Electroluminescence measurement. Electroluminescence spectrum measurement was conducted by direct-current meter (PWS2326, Tectronix) to provide bias voltage for the test device, and the electroluminescence emissions were recorded by the fluorescence spectrometer (KYMERA-328I-B2, Andor technology LTD).

EQE_{EL} measurement. The EQE_{EL} was recorded with an in-house-built system comprising a standard silicon photodiode 1010B, Keithley 2400 source meter (for supplying voltages and recording injected currents), and Keithley 6482 picoammeter (for measuring the emitted light intensity).

Highly sensitive EQE (s-EQE) The halogen light source (LSH-75, Newport) passed through the monochromator (CS260- RG-3-MC-A, Newport) to form monochromatic light, which was focused on the device to generate electrical signals. Signals were finally collected by the front-end current amplifier (SR570, Stanford) and phase-locked amplifier (Newport). A corrected silicon solar cell (S1337-1010BR) was used as a standard detector.

Transient absorption spectroscopy (TA) For femtosecond transient absorption

spectroscopy, the fundamental output from Yb:KGW laser (1030 nm, 220 fs Gaussian fit, 100 kHz, Light Conversion Ltd) was separated to two light beam. One was introduced to NOPA (ORPHEUS-N, Light Conversion Ltd) to produce a certain wavelength for pump beam (here we use 550 and 750 nm, 30 fs pulse duration), the other was focused onto a YAG plate to generate white light continuum as probe beam. The pump and probe overlapped on the sample at a small angle less than 10°. The transmitted probe light from sample was collected by a linear CCD array.

Impedance Spectroscopy (IS) measurement

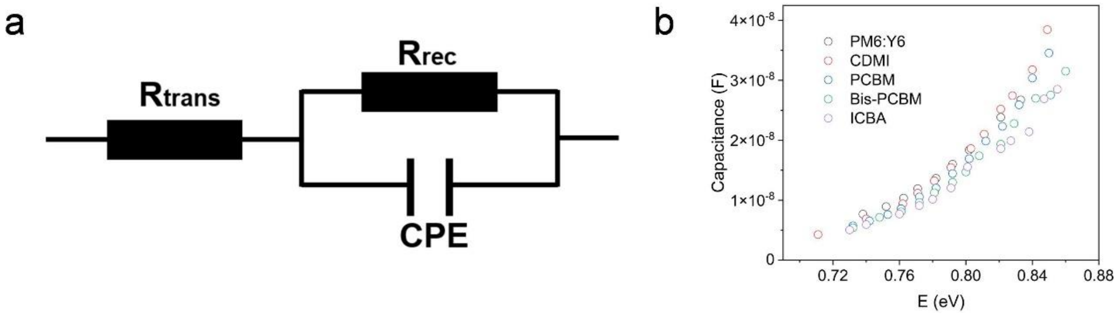

a, The equivalent circuit for impedance spectroscopy fitting. b, Chemical capacitance of the devices with binary and ternary BHJ layer extracted from impedance spectra. The method of obtaining DOS through IS is as reported in the literature. [1,2,3,4] Impedance measurements were carried out by illumination with a 1.5G illumination source (1000 W m^{-2}) using a Solar Simulator. The illumination intensity was tuned by filters. Impedance spectra were measured for different light intensities by applying a small voltage perturbation (10 mV rms) at frequencies from 8 MHz to 4 Hz, for different bias voltages. To measure in open circuit voltage conditions a bias voltage equals to V_{oc} at each light intensity was applied. The original data measured at different voltages are shown in Figure S29. These measurements were performed with LCR meeter equipped with a frequency analyzer module, always at room temperature. The equivalent circuit has been broadly used in bulk heterojunction organic photovoltaic devices.[1,2,3,4] $R_{internal}$ are internal (bulk) resistance. R_{rec} and CPE are the recombination resistance and the chemical capacitance, respectively. R_{rec} physically models the recombination paths through the device. CPE relates with the accumulation of photogenerated electrons and holes in the quasi-Fermi levels for electrons (E_{Fn}) and holes (E_{Fp}). The chemical capacitance (CPE) were extracted from the low-frequency region.[5,6] It has been reported that the chemical capacitance follows the shape of the electron DOS (g_n) as: [1,2,3,4]

$$C_\mu^n = Lq^2 g_n(E_{Fn})$$

Then the DOS results are fitted by exponential fitting to extract the DOS information.

Transient photovoltage (TPV) and photocurrent (TPC) measurement. The lifetime of carriers can be measured by the transient photovoltage measurement. The background illumination was provided by a normal LED light source, and pulsed light was provided by arbitrary wave generator. The photovoltage traces were registered by

the oscilloscope. The photocurrente traces were registered with the resistance of 50 Ω, switching open-circuit mode to short-circuit mode. The integrated TPC signal provides a measure of the total charge generated by the laser pulse (ΔQ). Empirically, the differential capacitance values are found to follow the exponential dependence on the open-circuit voltage given by $C = \frac{\Delta Q}{\Delta V} = C_0 exp(\gamma V_{OC}) + D$, and so the charge-carrier density as a function of V_{OC} is given by treating the device as a parallel-plate capacitor and integrating with respect to voltage, as $n = \frac{1}{Aed}\int_{-\infty}^{Voc} Coexp(\gamma V)dV$, where A is the active layer area, and d is the active layer thickness.

Supporting tables

Table S1. Crystallization analysis for binary and ternary films.

BHJ Types	Y6 (11-1) Area (a.u.)	Y6 (11-1) CCL (A)	PM6 (100) Area (a.u.)	PM6 (100) CCL (A)	Lamellar Area (a.u.)	pi-pi Area (a.u.)	Amorphous Area (a.u.)	Amorphous d-spacing (A)
PM6:Y6	48.68	113.03	205.87	63.08	240.94	589.69	223.81	4.58
T-C_{61}DMI	43.91	92.93	220.56	68.42	254.76	633.92	246.99	4.33
T-PC_{61}BM	45.74	104.89	228.74	69.54	264.81	647.32	242.46	4.42
T-Bis-PC_{61}BM	36.45	65.68	243.81	73.84	214.88	518.24	276.21	4.69
T-IC_{61}BA	30.82	77.47	250.55	75.12	220.92	530.73	271.74	4.75

Table S2. Lifetime of the hole transfer (biexponential fitting) and electron transfer (Single exponential fitting) process in blended films.

Blend	τ_1(ps)	τ_2(ps)	τ_n(ps)
PM6:Y6	0.317±0.006	13.89±0.572	89.42±3.37
T-C_{61}DMI	0.256±0.005	8.69±0.428	80.41±1.59
T-PC_{61}BM	0.274±0.008	10.54±0.433	83.45±2.14
T-Bis-PC_{61}BM	0.349±0.009	11.77±0.449	85.67±2.33
T-IC_{61}BA	0.358±0.009	12.83±0.506	87.89±3.25

Table S3. Results of density of state and FTPS-EQE by exponential fitting

Blends	E_t [meV]	E_U[meV]	E_{LUMO}[eV]	N_t[10^{20}cm^{-3}]
PM6:Y6	71.2±1.6	24.5±0.1	1.384	1.02±0.12
T-C_{61}DMI	63.6±1.1	21.3±0.2	1.381	1.90±0.21
T-PC_{61}BM	64.9±0.6	22.3±0.2	1.380	1.73±0.14
T-Bis-PC_{61}BM	72.9±2.8	25.7±0.3	1.402	0.82±0.09
T-IC_{61}BA	73.6±3.2	27.8±0.2	1.398	0.63±0.06

Table S4. Detailed electron structure of binary and ternary devices.

BHJ Types	Et [meV]	E_U [meV]	n [m^{-3}]	τ [μs]	τ_1 [ps]	τe [ps]	N_t [10^{20}cm^{-3}]
PM6:Y6	71.2	24.5	1.68	2.19	0.32	89.42	1.02
T-C_{61}DMI	63.6	21.8	2.26	3.17	0.25	80.41	1.9
T-PC_{61}BM	64.9	22.3	2.05	2.9	0.27	83.45	1.73
T-Bis-PC_{61}BM	72.9	25.7	1.25	1.95	0.35	85.67	0.82
T-IC_{61}BA	73.6	27.8	0.977	1.67	0.36	87.89	0.63

Supporting Figures

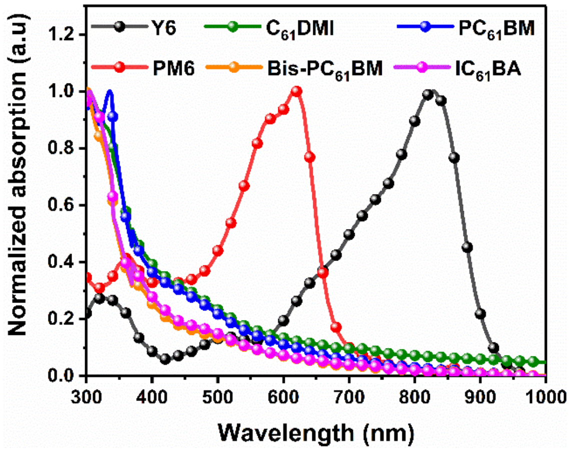

Figure S1. UV-vis absorption spectroscopy of pure films.

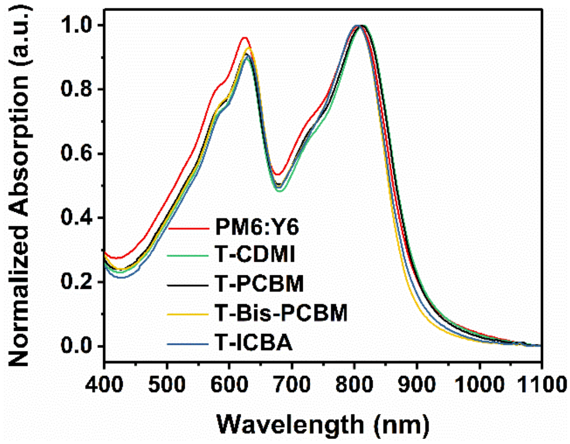

Figure S2. UV-vis absorption spectroscopy of binary and ternary films.

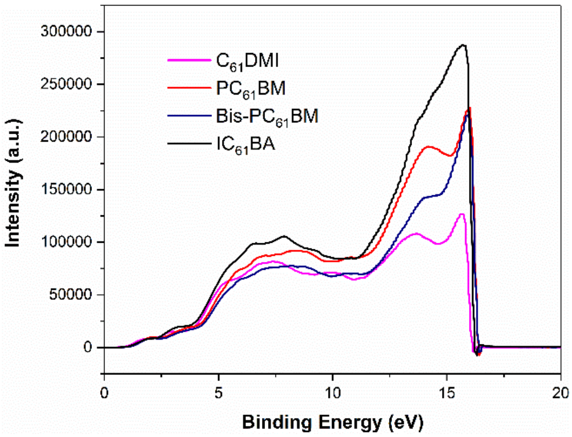

Figure S3. Ultraviolet photoelectron spectroscopy of fullerene derivatives.

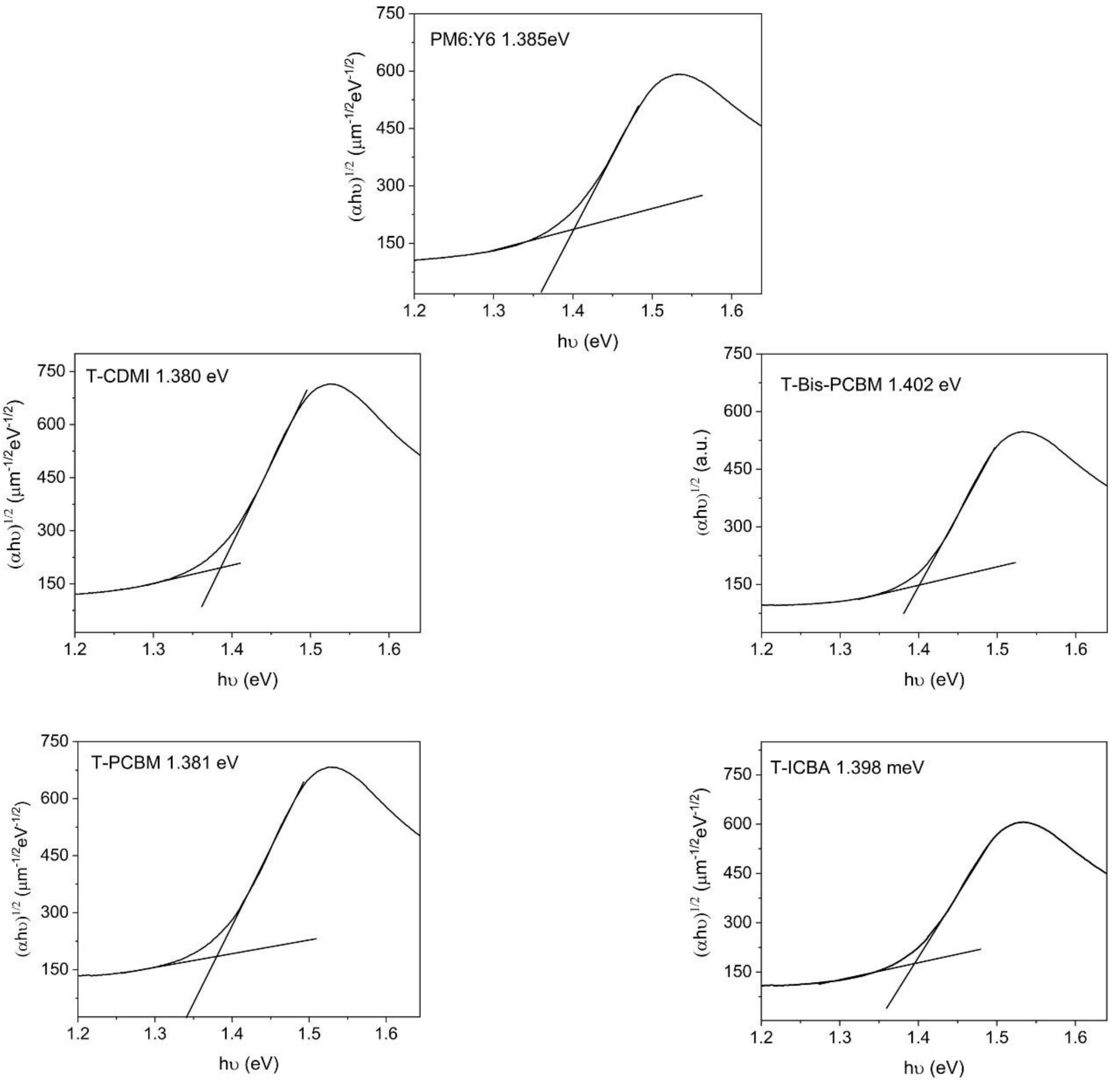

Figure S4. The optical bandgap of binary and ternary films.

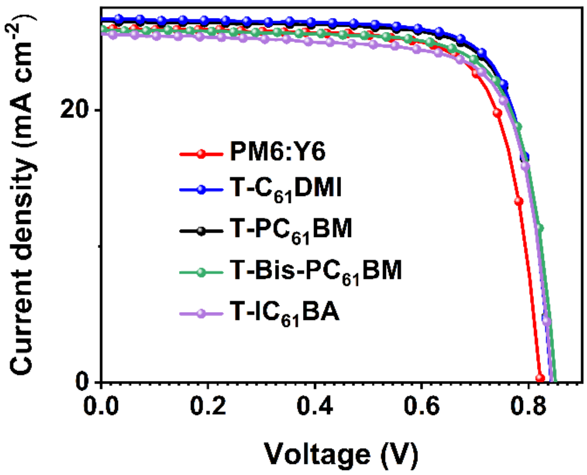

Figure S5. J-V curves of PM6:Y6 based binary and ternary devices.

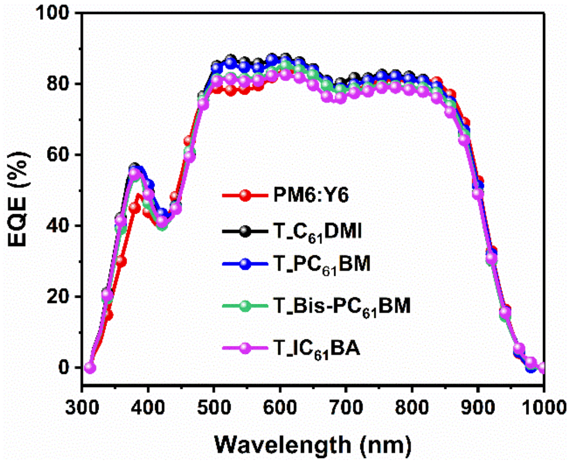

Figure S6. EQE curves of PM6:Y6 based binary and ternary devices.

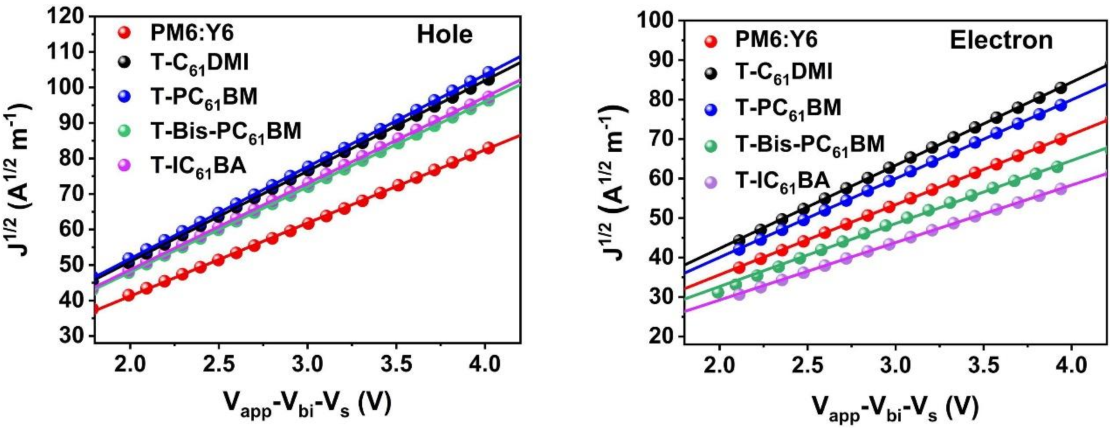

Figure S7. *J*-*V* characteristics of (a) hole-only and (b) electron-only devices for the measurement of the charge carrier transport in binary and ternary blends by the SCLC method.

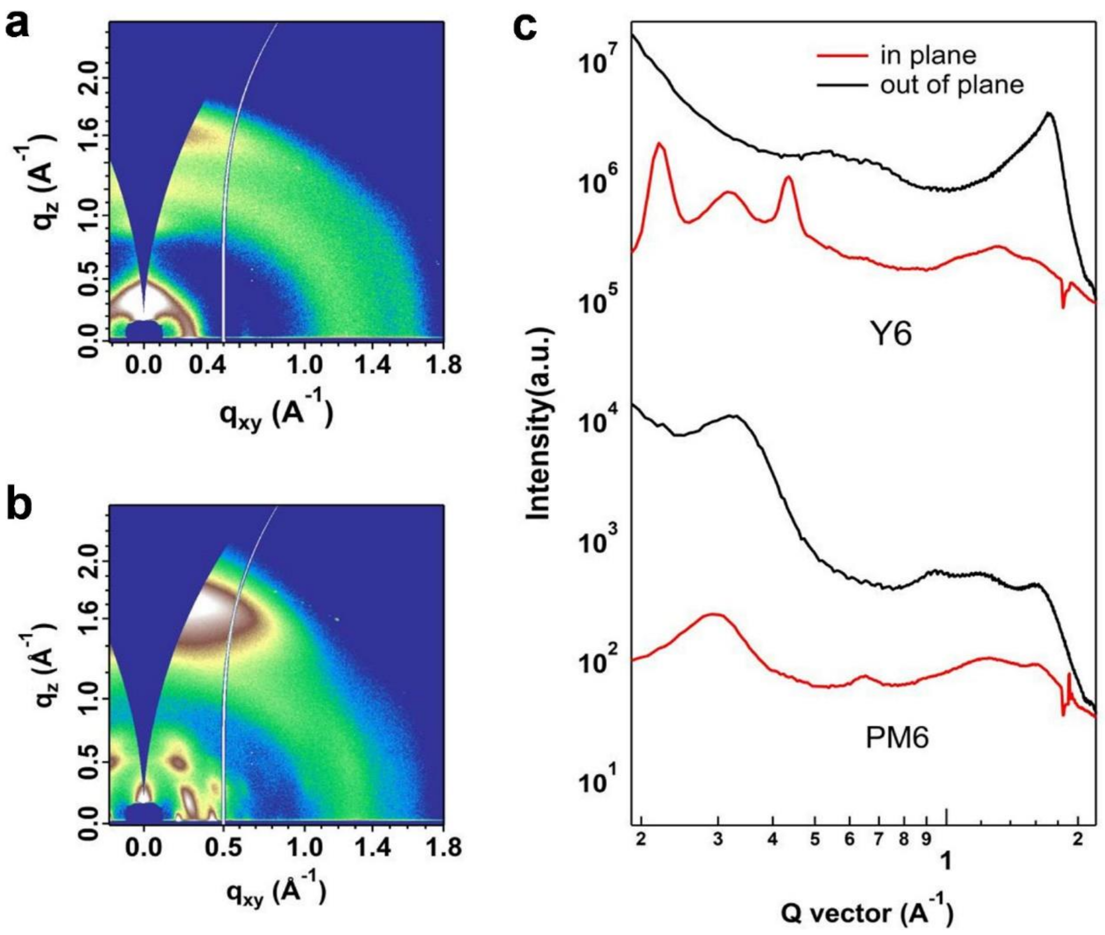

Figure S8. 2D GIWAXS patterns for a) pure Y6 and b) pure PM6 films and c) their Scattering profiles for pure films of PM6 and Y6.

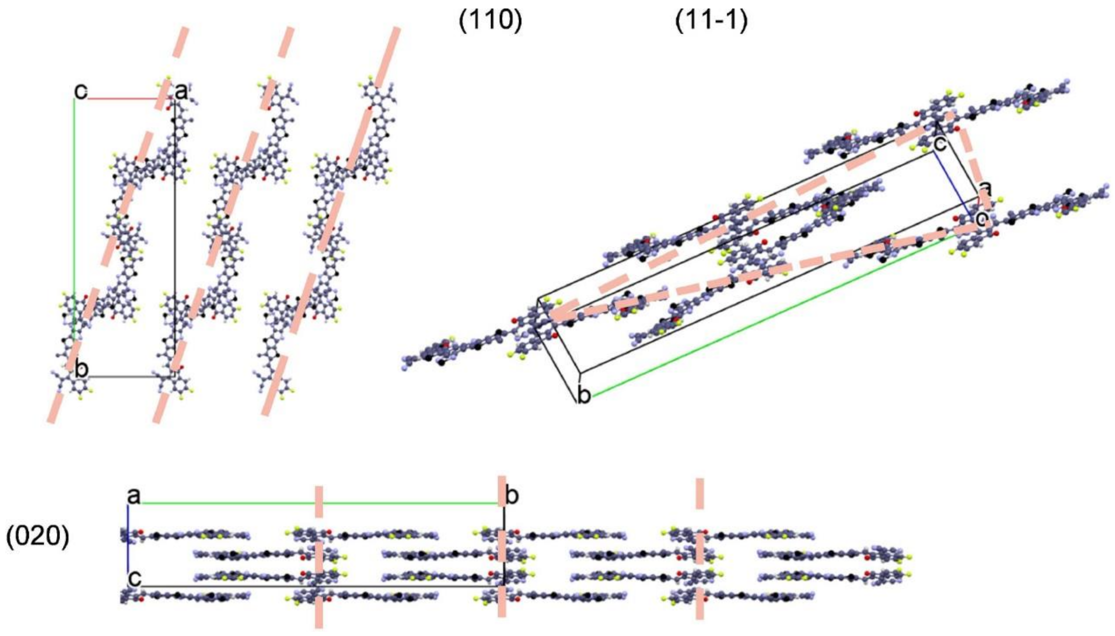

Figure S9. Stacking diagram of (110), (11-1), (020) crystal plane of Y6

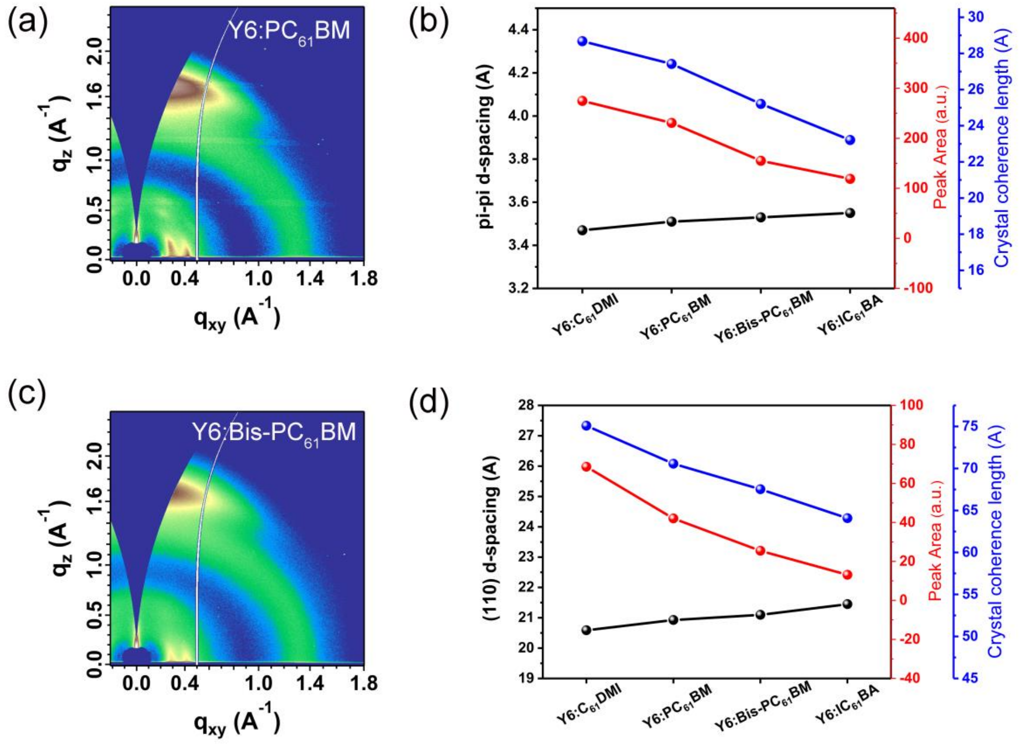

Figure S10. 2D GIWAXS patterns for a) Y6:PC_{61}BM and b) Y6:Bis-PC_{61}BM films and d-spacing, peak area and crystal coherence length of c) pi-pi stacking and d) (110) orientation of PM6: different fullerenes films.

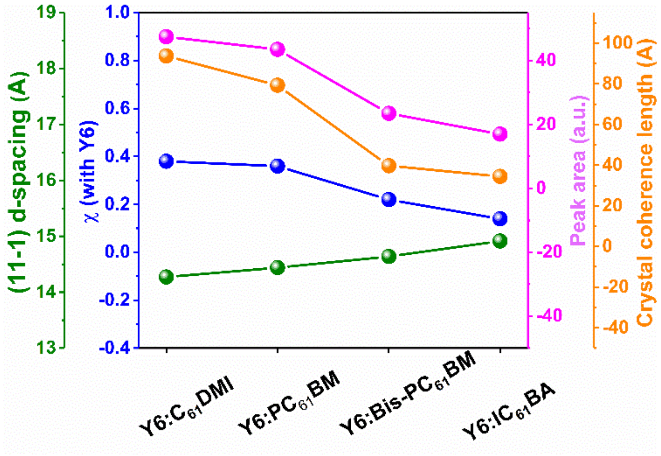

Figure S11..χ for Y6 with different FAs and The d-spacing, peak area and crystal coherence length of (11-1) orientation for Y6: different FAs films.

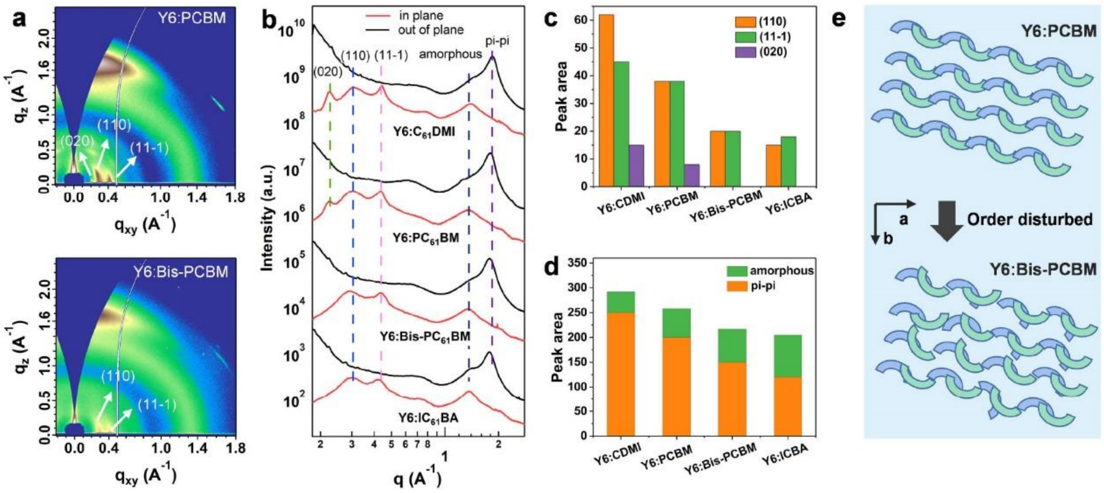

Figure S12. a 2D GIWAXS patterns for Y6:$PC_{61}BM$ and Y6:Bis-$PC_{61}BM$. **b** Scattering profiles for Y6:FAs films. **c** peak area for (110), (11-1), (020) **d** amorphous peak and pi-pi stacking peak. **e** Diagrams of Y6 molecular arrangement in different Y6:FAs films.

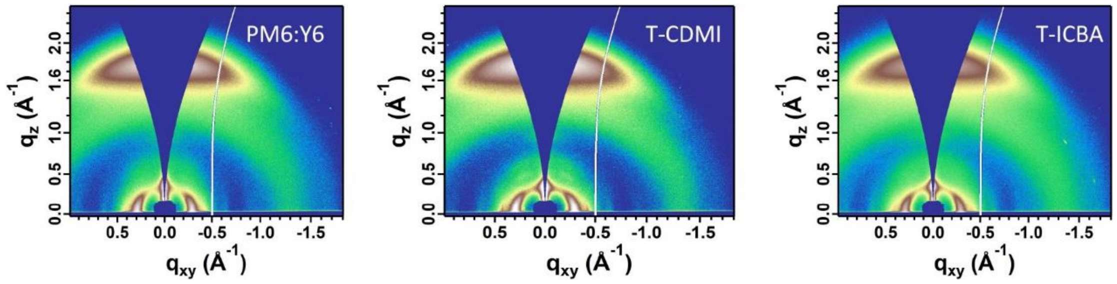

Figure S13. 2D GIWAXS patterns for PM6:Y6, T-$PC_{61}BM$ and T-Bis-$PC_{61}BM$ films.

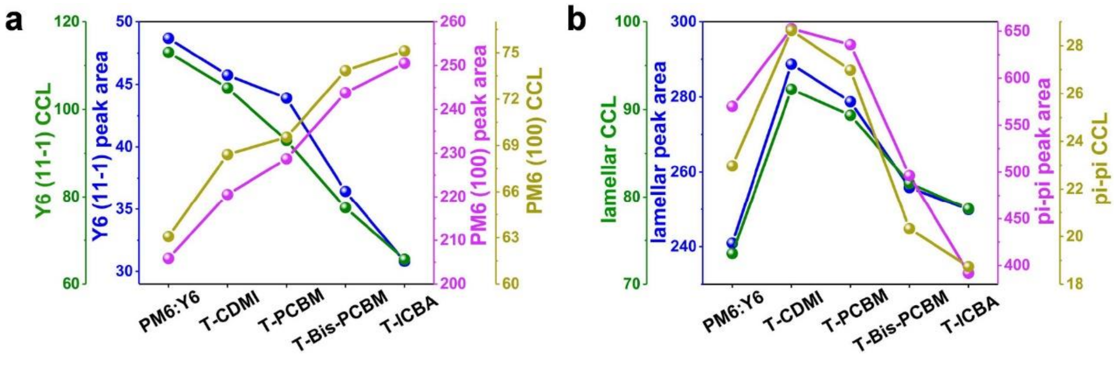

Figure S14. Peak area and CCL for a) Y6(11-1) and PM6 (100) peaks and b) lamellar and pi-pi stacking.

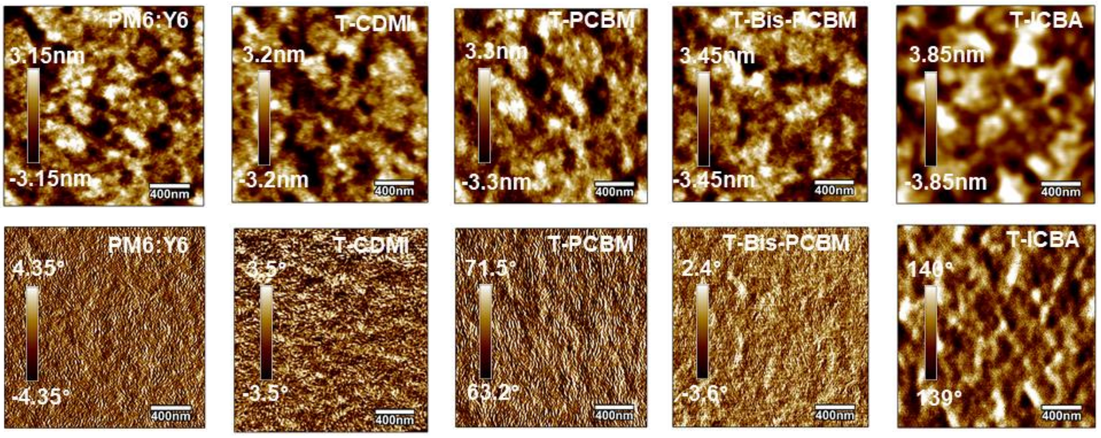

Figure S15. The height and phase images for binary and ternary films.

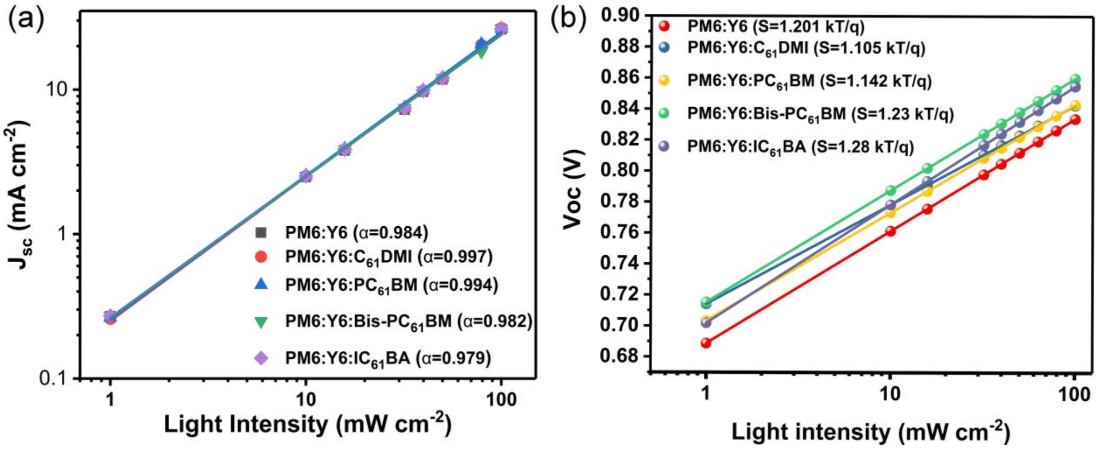

Figure S16. (a) J_{SC} and (b) V_{OC} dependence on light intensity of binary and ternary devices.

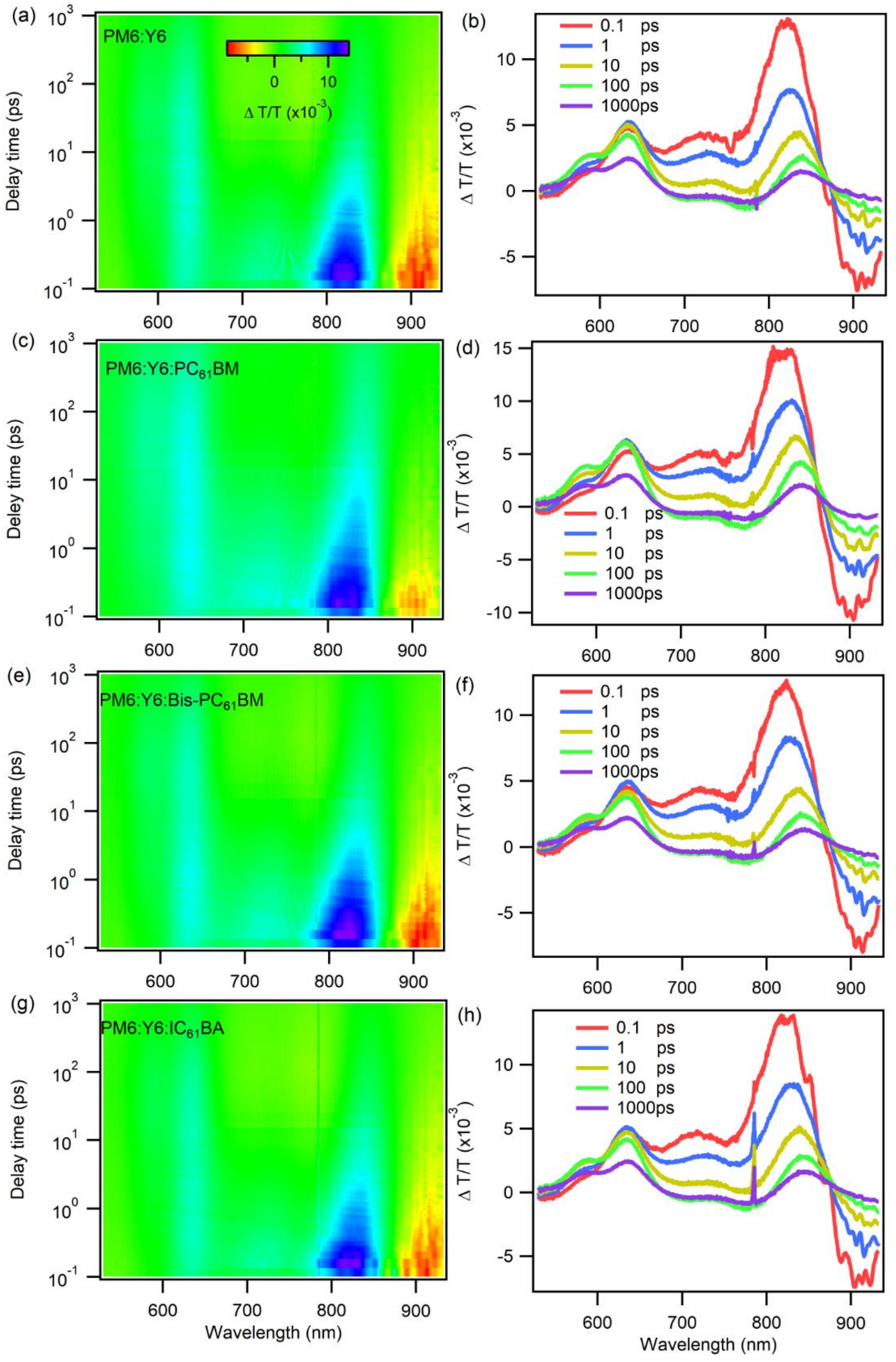

Figure S17. Color plot of fs transient absorption spectra of (a) PM6:Y6, (c) PM6:Y6:$PC_{61}BM$, (e) PM6:Y6:Bis-$PC_{61}BM$ and (f) PM6:Y6:$IC_{61}BA$ blend film at indicated delay times under 750 nm excitation with a fluence below 10 μJ/cm^2. (b)

Representative fs Transient absorption spectra of (b) PM6:Y6, (d) PM6:Y6:$PC_{61}BM$, (f) PM6:Y6:Bis-$PC_{61}BM$ and (h) PM6:Y6:$IC_{61}BA$ blend film at indicated delay time.

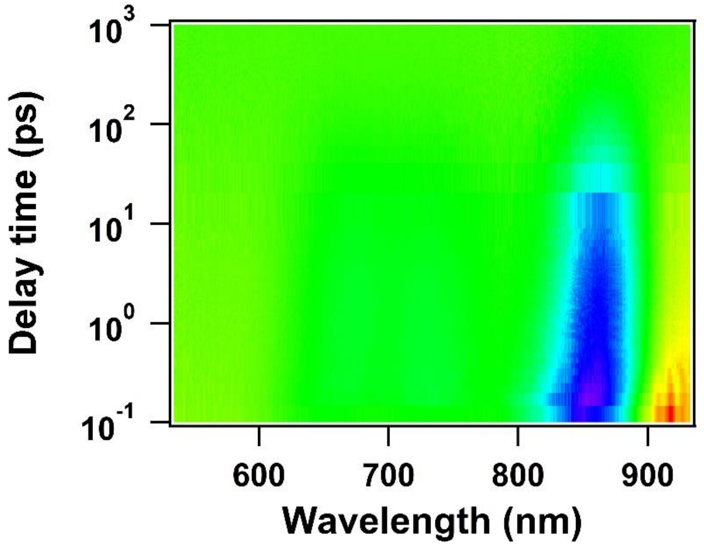

Figure S18. Y6 blend films at indicated delay times under 750 nm excitation with a fluence below 10 μJ/cm^2.

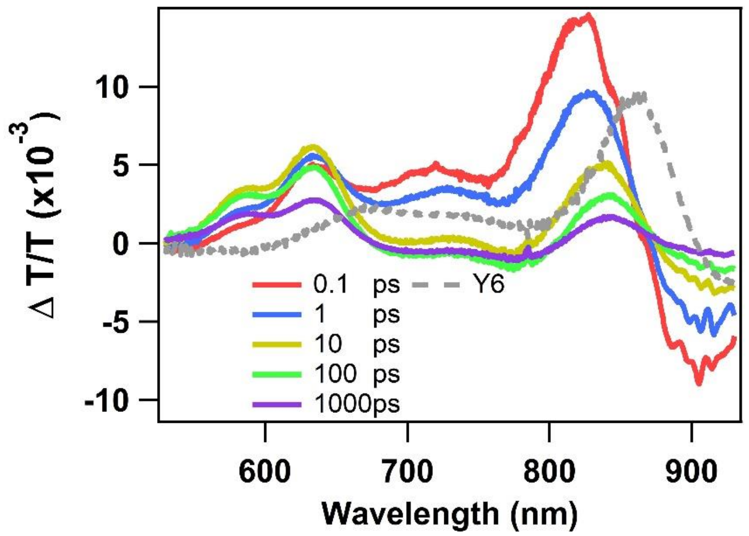

Figure S19. Representative fs Transient absorption spectra of PM6:Y6:$IC_{61}DMI$ blend film at indicated delay time. Gray dots: TA spectrum of Y6 blend film excited at 750 nm.

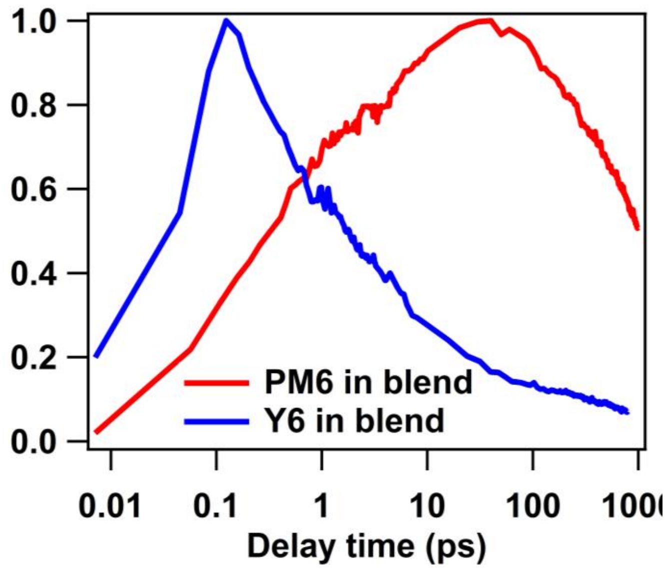

Figure S20. TA kinetics of PM6:Y6 based binary and ternary blend film indicating of hole transfer process.

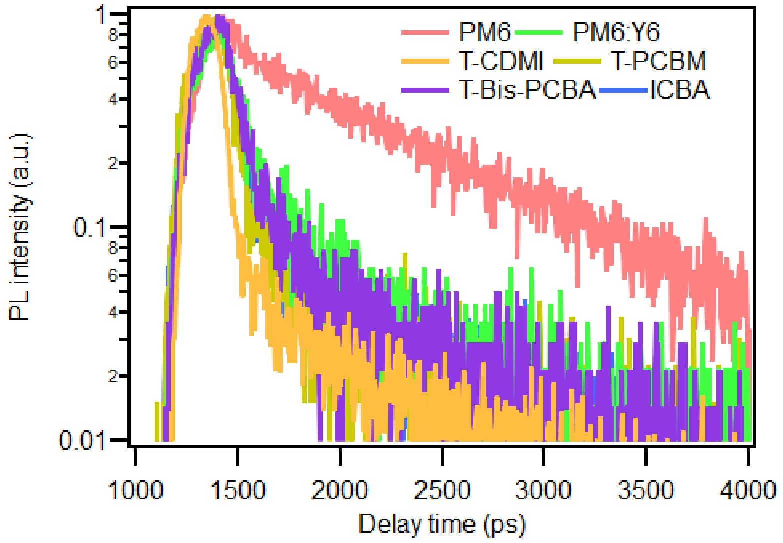

Figure S21 TRPL of neat PM6 film and PM6:Y6, PM6:Y6:$PC_{61}BM$, PM6:Y6:PCBM, PM6:Y6:Bis-$PC_{61}BM$, PM6:Y6:$IC_{61}BA$ blend film excited at 480 nm probe at 500-700 nm indicating of electron transfer process.

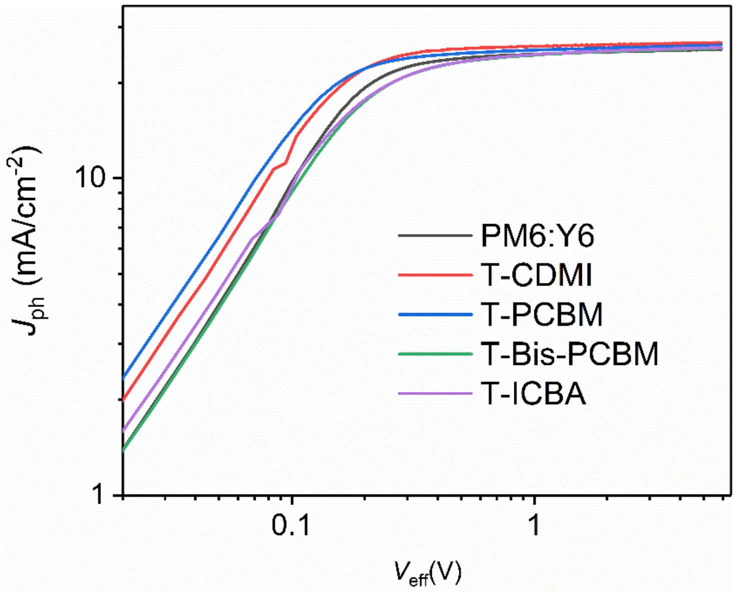

Figure S22. J_{ph}-V_{eff} curves.

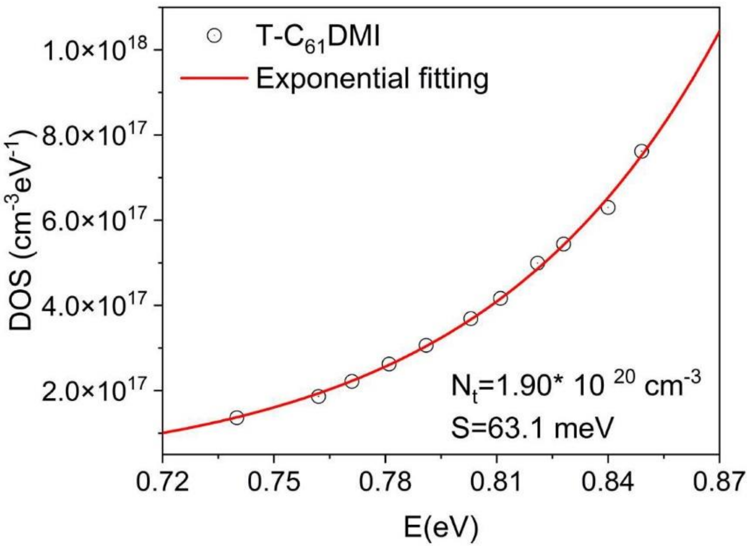

T-C61DMI
Exponential fitting
DOS (cm-3eV-1)
1.0×1018
8.0×1017
6.0×1017
4.0×1017
2.0×1017
Nt=1.90* 10 20 cm-3
S=63.1 meV
0.72
0.75
0.78
0.81
0.84
0.87
E(eV)

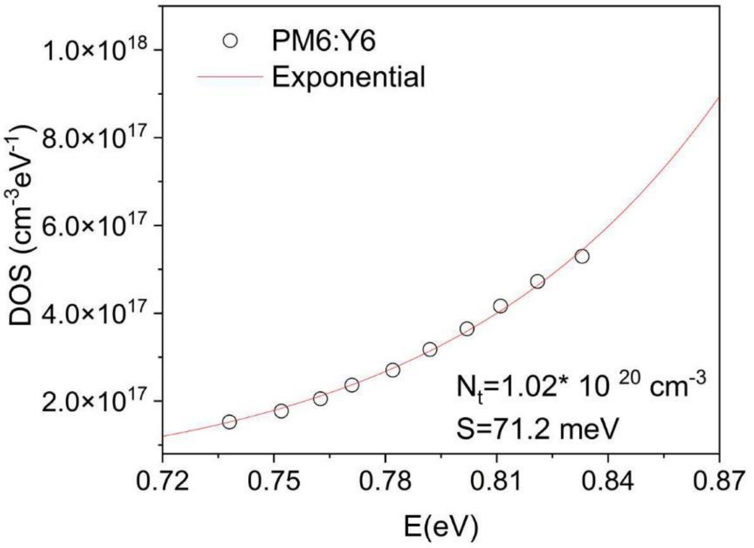

PM6:Y6
Exponential
DOS (cm-3eV-1)
1.0×1018
8.0×1017
6.0×1017
4.0×1017
2.0×1017
Nt=1.02* 10 20 cm-3
S=71.2 meV
0.72
0.75
0.78
0.81
0.84
0.87
E(eV)

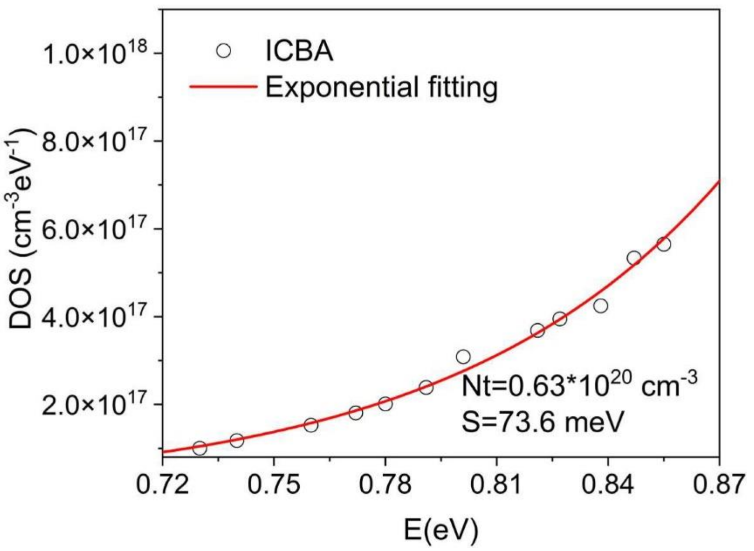

ICBA
Exponential fitting
DOS (cm-3eV-1)
1.0×1018
8.0×1017
6.0×1017
4.0×1017
2.0×1017
Nt=0.63*1020 cm-3
S=73.6 meV
0.72
0.75
0.78
0.81
0.84
0.87
E(eV)

Figure S23 DOS fitting curves for PM6:Y6;T-CDMI;T-ICBA devices.

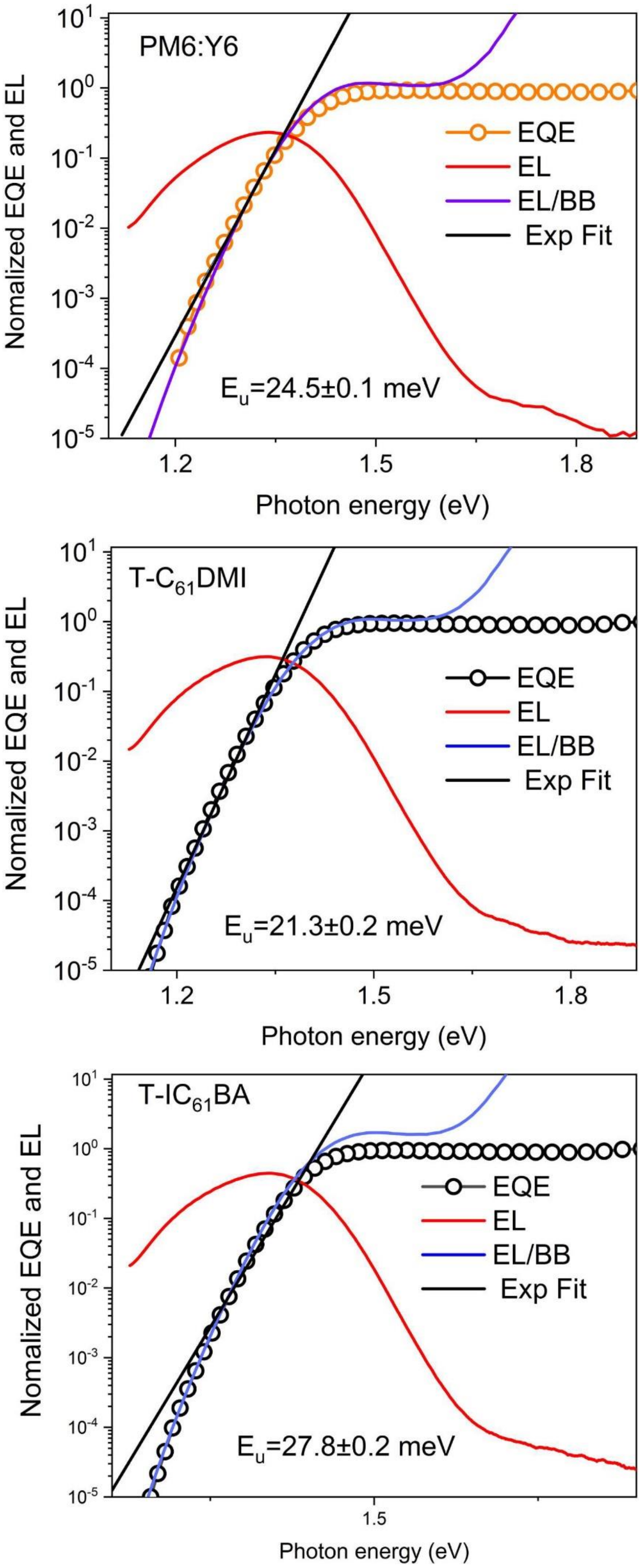
PM6:Y6
EQE
EL
EL/BB
Exp Fit
E_u=24.5±0.1 meV
Nomalized EQE and EL
Photon energy (eV)
T-C_{61}DMI
EQE
EL
EL/BB
Exp Fit
E_u=21.3±0.2 meV
Nomalized EQE and EL
Photon energy (eV)
T-IC_{61}BA
EQE
EL
EL/BB
Exp Fit
E_u=27.8±0.2 meV
Nomalized EQE and EL
Photon energy (eV)

Figure S24 E_U and E_{CT} fitting curves for PM6:Y6;T-CDMI and T-ICBA devices.

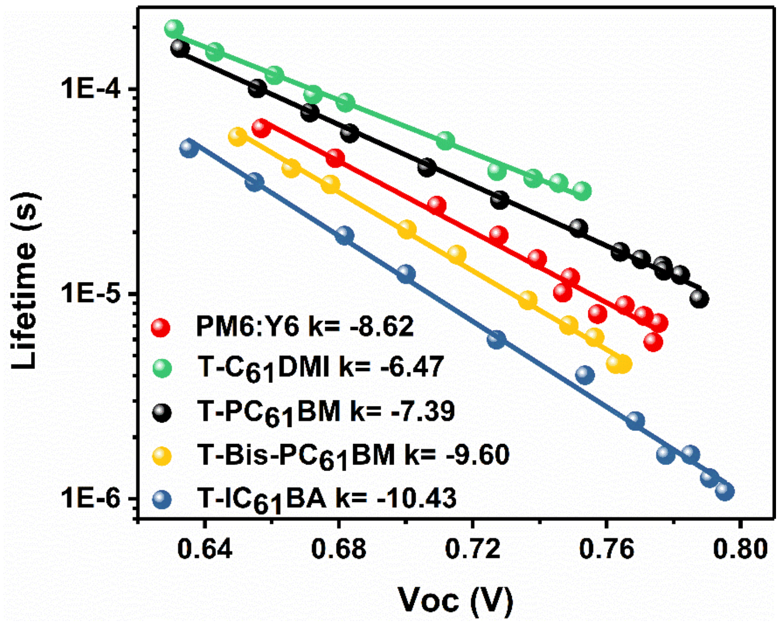

Figure S25. Charge lifetime in the devices as a function of charge density.

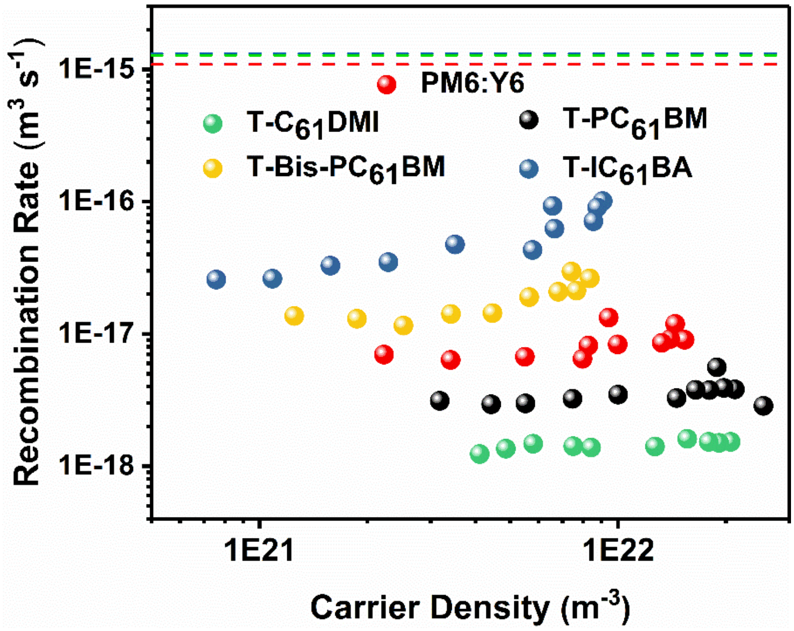

Figure S26. Measured nongeminate recombination rate coefficient for devices. The dash lines represent Langevin recombination rate coefficient for different devices.

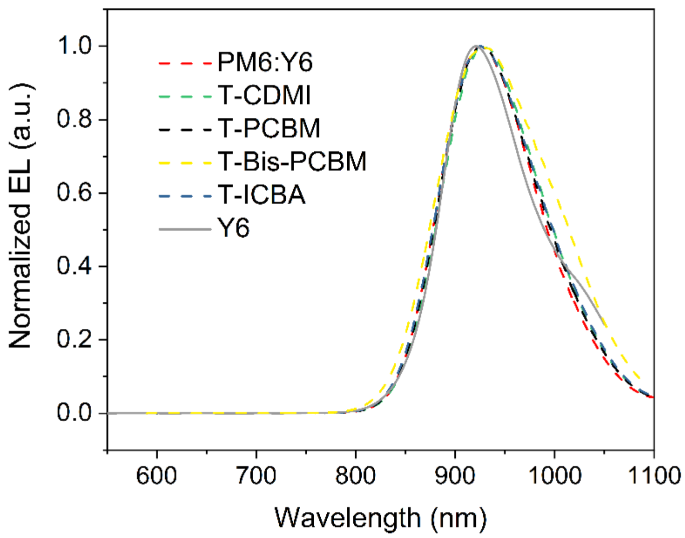

Figure S27. EL spectra of Y6,binary and ternary blends.

Appendix: Summary of Certificate

NIM Certificate No.: GXgf2019-00971

Client: SHANGHAI JIAO TONG UNIVERSITY

Sample: Organic Solar Cells

Type/Model: Non-fullerene polymer solar cell

DUT S/N：L33#-M2-F-01

Manufacturer：SHANGHAI JIAO TONG UNIVERSITY

Date of Test: 09/20/2019

Temperature Sensor/Control System: None

Environmental conditions: (23.6±1)℃, RH (53.2±2)%

Mask: Aperture area of 3.153 mm^2 (Certificate No.: CDjc2019-5610).

The calibration has been conducted by the PV Metrology Lab of NIM (National Institute of Metrology, China). Measurement of irradiance intensity and all other measurements are traceable to the International System of Units (SI). The performance parameters reported in this certificate apply only at the time of the test for the sample.

Area (mm^2)	I_{sc}(mA)	V_{oc}(V)	P_{max}(mW)
3.153	0.826	0.845	0.540
I_{max}(mA)	V_{max}(V)	FF (%)	η (%)
0.750	0.720	77.3	17.1

I-V Characterization Methods:

IEC60904-1 2006: Measurement of photovoltaic current-voltage characteristics

JJF 1622-2017: Calibration Specification of Solar Cells：Photoelectric Properties

Secondary Reference Cell:

Device S/N: 81#; Device Material: Mono-Si

Solar Simulator:

Classification: AAA (Double-light source: Xeon and Halogen);

Total irradiance: 1000 W/m^2 based on I_{sc} of the above Secondary Reference Cell.

Figure S28. Verification report for PM6:Y6:CDMI devices.

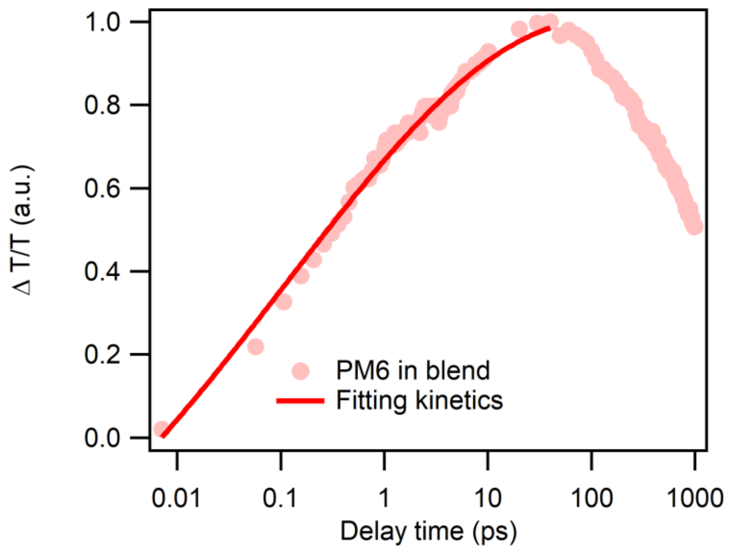

Figure S29. GSB kinetics fitting

References:

[1] Fabregat-Santiago F, Garcia-Belmonte G, Mora-Sero I, et al. Characterization of nanostructured hybrid and organic solar cells by impedance spectroscopy[J]. Physical chemistry chemical physics, 2011, 13(20): 9083-9118.

[2] Shao, Y.; Yuan, Y.; Huang, J., Correlation of energy disorder and open-circuit voltage in hybrid perovskite solar cells. Nature Energy 2016, 1 (1), 15001.

[3] Garcia-Belmonte, G.; Boix, P. P.; Bisquert, J.; Lenes, M.; Bolink, H. J.; La Rosa, A.; Filippone, S.; Martín, N., Influence of the intermediate density-of-states occupancy on open-circuit voltage of bulk heterojunction solar cells with different fullerene acceptors. The Journal of Physical Chemistry Letters 2010, 1 (17), 2566-2571.

[4] Garcia-Belmonte, G.; Boix, P. P.; Bisquert, J.; Sessolo, M.; Bolink, H. J., Simultaneous determination of carrier lifetime and electron density-of-states in P3HT:PCBM organic solar cells under illumination by impedance spectroscopy. Solar Energy Materials and Solar Cells 2010, 94 (2), 366-375.

[5] Brus, V. V., Proctor, C. M., Ran, N. A., Nguyen, Thuc-Quyen (2016). Capacitance Spectroscopy for Quantifying Recombination Losses in Nonfullerene Small-Molecule

Bulk Heterojunction Solar Cells. Adv. Energy Mater., 6: 1502250. doi: 10.1002/aenm.201502250

[6] Vollbrecht, J., Brus, V. V., Ko, S.-J., Lee, J., Karki, A., Cao, D. X., Cho, K., Bazan, G. C., Nguyen, T.-Q., Quantifying the Nongeminate Recombination Dynamics in Nonfullerene Bulk Heterojunction Organic Solar Cells. Adv. Energy Mater. 2019, 9, 1901438. https://doi.org/10.1002/aenm.201901438
